# Measurement of very low-molecular weight metabolites by traveling wave ion mobility and its use in human urine samples

**DOI:** 10.1016/j.jpha.2023.12.011

**Published:** 2023-12-16

**Authors:** Alongkorn Kurilung, Suphitcha Limjiasahapong, Khwanta Kaewnarin, Pattipong Wisanpitayakorn, Narumol Jariyasopit, Kwanjeera Wanichthanarak, Sitanan Sartyoungkul, Stephen Choong Chee Wong, Nuankanya Sathirapongsasuti, Chagriya Kitiyakara, Yongyut Sirivatanauksorn, Sakda Khoomrung

**Affiliations:** a Siriraj Center of Research Excellent in Metabolomics and Systems Biology (SiCORE-MSB), Faculty of Medicine Siriraj Hospital, Mahidol University, Bangkok, 10700, Thailand; b Siriraj Metabolomics and Phenomics Center, Faculty of Medicine Siriraj Hospital, Mahidol University, Bangkok, 10700, Thailand; c Department of Biomedical Informatics, University of Arkansas for Medical Sciences, Little Rock, AR, 72205, USA; d SingHealth Duke-NUS Institute of Biodiversity Medicine, National Cancer Centre Singapore, 168583, Singapore; e Waters Pacific Pte., Ltd., Singapore, 117528, Singapore; f Program in Translational Medicine, Faculty of Medicine Ramathibodi Hospital, Mahidol University, Bangkok, 10400, Thailand; g Chakri Naruebodindra Medical Institute, Faculty of Medicine Ramathibodi Hospital, Mahidol University, Samut Prakan, 10540, Thailand; h Department of Medicine, Ramathibodi Hospital, Mahidol University, Bangkok, 10400, Thailand; i Department of Biochemistry, Faculty of Medicine Siriraj Hospital, Mahidol University, Bangkok, 10700, Thailand; j Center of Excellence for Innovation in Chemistry (PERCH-CIC), Faculty of Science, Mahidol University, Bangkok, 10400, Thailand

**Keywords:** Very low-molecular-weight metabolites, Traveling wave ion mobility, Collision cross-section, Human urine

## Abstract

The collision cross-sections (CCS) measurement using ion mobility spectrometry (IMS) in combination with mass spectrometry (MS) offers a great opportunity to increase confidence in metabolite identification. However, owing to the lack of sensitivity and resolution, IMS has an analytical challenge in studying the CCS values of very low-molecular-weight metabolites (VLMs ≤ 250 Da). Here, we describe an analytical method using ultrahigh-performance liquid chromatography (UPLC) coupled to a traveling wave ion mobility-quadrupole-time-of-flight mass spectrometer optimized for the measurement of VLMs in human urine samples. The experimental CCS values, along with mass spectral properties, were reported for the 174 metabolites. The experimental data included the mass-to-charge ratio (*m*/*z*), retention time (RT), tandem MS (MS/MS) spectra, and CCS values. Among the studied metabolites, 263 traveling wave ion mobility spectrometry (TWIMS)-derived CCS values (^TW^CCS_N2_) were reported for the first time, and more than 70% of these were CCS values of VLMs. The ^TW^CCS_N2_ values were highly repeatable, with inter-day variations of <1% relative standard deviation (RSD). The developed method revealed excellent ^TW^CCS_N2_ accuracy with a CCS difference (ΔCCS) within ±2% of the reported drift tube IMS (DTIMS) and TWIMS CCS values. The complexity of the urine matrix did not affect the precision of the method, as evidenced by ΔCCS within ±1.92%. According to the Metabolomics Standards Initiative, 55 urinary metabolites were identified with a confidence level of 1. Among these 55 metabolites, 53 (96%) were VLMs. The larger number of confirmed compounds found in this study was a result of the addition of ^TW^CCS_N2_ values, which clearly increased metabolite identification confidence.

## Highlights

• Reduce bias voltages and WH and increase WV enabled the measurement of VLMs by TWIMS.
• A total of 263 ^TW^CCS_N2_ were first reported.
• An experimental IM-MS library of 174 standards with *m*/*z*, RT, MS/MS, and CCS was created.
• The application of the IM-MS library successfully identified 55 metabolites in human urine.

## Graphical abstract

**Figure:**
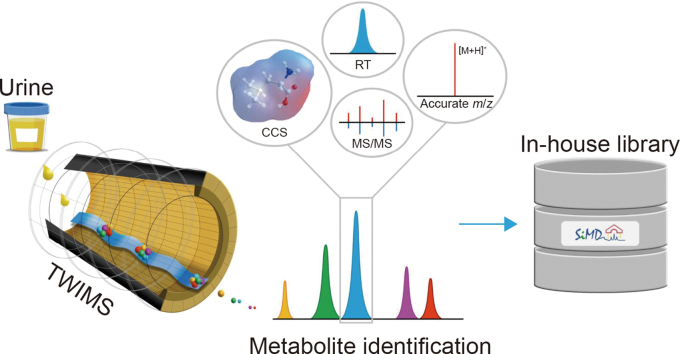

## 1 Introduction

Low-molecular-weight metabolites and very low-molecular-weight metabolites (VLMs) are small molecules, which are characterized by molecular weight below 900 and 250 Da, respectively [1]. Measuring these small metabolites in the biological samples, especially VLMs, is still a challenge as they have an enormous number, a vast diversity of chemical structures, and low concentrations [2]. These challenges can be addressed with the use of liquid chromatography (LC) with a high-resolution quadrupole-time-of-flight (QTOF) mass spectrometry (MS) in combination with ion mobility spectrometry (IMS), which generates multi-dimensional separation with high selectivity for the metabolites [2,3].

IMS is a rapid gas-phase separation technology in which ions are separated based on their mobility through a neutral buffer gas (typically helium or nitrogen) under the influence of an electric field [4,5]. IMS not only presents an additional dimension of separation for MS but also provides a measurement of the rotationally averaged collision cross-section (CCS) [[3], [4], [5]]. The measured CCS value is influenced by the ion structure, the drift gas, the temperature of the ions, and the ratio between the electric field strength and the number density of the gas (*E*/*N*) [6]. CCS values are used as an orthogonal molecular descriptor to characterize various types of compounds [[7], [8], [9], [10], [11], [12], [13], [14]], along with mass-to-charge ratio (*m*/*z*), retention time (RT), and MS/MS spectra. The combination of such molecular descriptors has been demonstrated to resolve coeluting isomeric compounds in various complex matrices [5,[15], [16], [17]].

IMS has been used to characterize structural biomolecules in cells, biofluids, and tissues. For example, IMS was applied for targeted screening of small organic molecules [12], drugs [14], xenobiotics [18], and contaminants of emerging concern in urine samples [19]. IMS has been used to detect metabolites in human urine samples [20,21], with a restricted number of metabolites verified against reference standards. Since the use of IMS can increase peak capacity, it enhances the detection of low-concentration compounds by reducing interference from chemical noise [12]. Recently, IMS has been used to create experimental CCS values and incorporated into the CCS database to improve confidence in compound identification, covering various types of small molecules [18,[22], [23], [24]], lipids [25], peptides [24], and glycans [26]. Although the number of experimental CCS databases is increasing, the experimental CCS values for VLMs are still limited compared to other biomolecules [27]. This is partially because of the space charge effect in drift tube IMS (DTIMS) [28] or field-induced ion heating in traveling wave IMS (TWIMS) [7,29], both of which can cause to the poor ion transmission of these small molecules to the ion mobility (IM) chamber. It is known that TWIMS and field-asymmetric waveform IMS operate under higher electric field intensity compared with DTIMS [30,31]. Under such conditions, field-induced heating influences the dissociation of ions with effective temperature between 226.85 and 526.85 °C [29,32]. Additionally, ion heating may cause unwanted conformational changes, resulting in incorrect CCS measurements. To alleviate ion heating and improve ion transmission, reducing the bias voltage used to push ions into the mobility separation region, lowering the traveling wave height (WH), and increasing the wave velocity (WV) have been suggested [29,32]. The bias voltage helps accelerate ions against the pressure gradient between the trap and IM regions. This injection process can induce dissociation of labile VLM ions before they enter the IM chamber. To counter this, a helium cell at the entrance to the IMS region reduces ion energy through collisional cooling. To further assist the analysis of labile VLMs, the bias voltage must be kept lower than the default value of the instrumental setting; however, there is a trade-off between minimizing the dissociation of the most labile compounds, while also maximizing the transmission of the largest ions (least mobile) of interest [29,33]. On the other hand, WH and WV are directly and inversely proportional to the ion temperature, respectively. By lowering WH or increasing WV, ions are accelerated through the IM cell under high electric field intensity at higher speeds, which in turn decreases ion dissociation [29,32,33]. Although previous TWIMS studies employed a constant WH (40 V) and WV (approximately 600–650 m/s) for metabolomics analysis [12,13,34,35], there is no clear consensus on whether these settings provide optimal traveling wave (T-wave) conditions for all metabolites, especially VLMs, which tend to be more sensitive to heating-induced dissociation, as they have fewer bonds, and thus fewer degrees of freedom with which to dissipate excess internal vibrational energy, than larger molecules. Consequently, it is critical to properly evaluate the T-wave parameters to manage the actual temperature of the ions and to obtain good mobility separation [36].

In this study, we evaluated the measurement conditions for the analysis of VLMs using ultra-performance liquid chromatography coupled with TWIMS-QTOF-MS (UPLC/TWIMS-QTOF-MS). A total of 174 reference standards representing various chemical classes of metabolites were used for method development. The accuracy, precision, and effects of the biological matrix on the derived TWIMS-derived CCS values (^TW^CCS_N2_) values were investigated. The IM–MS properties, including *m*/*z*, RT, fragment ions (MS/MS), and CCS values, were constructed to an in-house library, which was subsequently incorporated into a metabolomics workflow to facilitate metabolite identification in human urine. This study demonstrates, for the first time, the optimized TWIMS settings for measuring VLMs in human urine and establishes an IM–MS library with 309 ^TW^CCS_N2_ that can facilitate the identification of small metabolites in human urine and other biological samples.

## 2 Material and methods

### 2.1 Chemicals, reagents, and standards

Acetonitrile, methanol, formic acid (Fisher Scientific, Geel, Belgium), ammonium formate (Thermo Fisher Scientific Inc., Ward Hill, MA, USA), and ammonium acetate (Loba Chemie, Mumbai, India) were high performance liquid chromatography (HPLC) grade. Ultra-pure water (18.2 MΩ‧cm, 25 °C, <10 ppb) was obtained using a Milli-Q purification system (Millipore, Molsheim, France). A total of 174 analytical standards were purchased from Sigma-Aldrich (Stuttgart, Germany) (see Supplementary data and Table S1 for further details). The Major Mix IMS/TOF Calibration Kit and leucine encephalin were purchased from Waters (Milford, MA, USA).

### 2.2 Ethic statement and sample preparation

This study was approved by the Ethical Clearance Committee on Human Rights Related to Research Involving Human Subjects, Faculty of Medicine, Ramathibodi Hospital, Mahidol University, Thailand (Approval No.: MURA2019/769). Human urine samples were prepared based on a previously described method with slight modification [37]. A total of 100 μL of pooled urine samples (*n* = 3) was diluted with 900 μL of acetonitrile:methanol (1:1, *V*/*V*), vortexed for 30 s, sonicated for 10 min, and stored at −20 °C for 1 h to facilitate protein precipitation. Subsequently, the samples were centrifuged at 13,000 *g* for 15 min. The supernatant (900 μL) was collected and kept at −80 °C until analysis.

### 2.3 UPLC/TWIMS-QTOF-MS analysis

Standards and urine samples were chromatographically separated by UPLC (ACQUITY UPLC, Waters) using an ACQUITY BEH HILIC column (2.1 mm × 100 mm, 1.7 μm; Waters). Separate acidic and basic chromatographic methods have been developed for positive and negative electrospray ionization (ESI), respectively, to accommodate the analysis of analytes with a wide range of polarities. Mobile phase A consisted of acetonitrile:water (95:5, *V*/*V*) and mobile phase B consisted of acetonitrile:water (50:50, *V*/*V*). Under acidic conditions, both mobile phases contained 10 mM ammonium formate and 0.125% formic acid (pH 3.0), and under basic conditions, both contained 10 mM ammonium acetate and 0.04% (*V*/*V*) ammonium hydroxide (pH 9.0). An injection volume of 5 μL and column temperature of 45 °C were used under both conditions. The chromatographic gradient was as follows: 0−1 min, 99% A; 1−10 min, 99%–65% A; 10−12 min, 65%–40% A; 12−15 min 40% A; and 15−20 min, re-equilibration at 99% A, with a flow rate of 0.4 mL/min.

The UPLC system was coupled to a TWIMS-QTOF-MS system (Synapt G2-Si, Waters, Manchester, UK) with an ESI source. In the positive ESI mode (ESI^+^), the following parameters were applied: capillary voltage 2.5 kV; cone voltage 30 V; source temperature 100 °C; and desolvation temperature 200 °C. In the negative ESI mode (ESI^−^), the following conditions were used: capillary voltage 2.0 kV; cone voltage 40 V; source temperature 100 °C; and desolvation temperature 200 °C. For the TWIMS settings, the optimized parameters for the ESI^+^ mode were as follows: nitrogen flow rate 90 mL/min; WV 800 m/s; WH 30 V; trap bias 35 V; and helium bias 30 V. For the ESI^−^ mode, the following optimized parameters were used: nitrogen flow rate at 90 mL/min; WV 1000 m/s; WH 30 V; trap bias at 35 V; and helium bias at 30 V. The TWIMS-QTOF-MS was mass and CCS calibrated using the Major Mix IMS/TOF Calibration solution, and real-time single-point calibration correction was performed using leucine enkephalin as the reference LockMass and LockCCS to maintain mass and CCS accuracy during the long analytical acquisitions.

Data were collected using the data-independent high definition MS^E^ (HDMS^E^) mode with a 0.2 s scan time and mass range of *m*/*z* 50−1000. In the HDMS^E^ mode, the quadrupole is non-selective (radio frequency (RF) only), and the collision energy (CE) alternates between a low-energy function (CE 4 eV) to monitor intact precursor ions and a high-energy function (CE ramp 20–40 eV) to observe the dissociation product ions. Argon (≥99.999%) was used as the collision gas for collision-induced dissociation (CID). The TOF analyzer was operated in sensitivity mode, which provided a resolving power of approximately 10,000 full width at half maximum (FWHM).

### 2.4 Precision, accuracy, and matrix effect on TWIMS-derived CCS values

To determine the precision of the ^TW^CCS_N2_, the experiments were carried out using a set of representative standard metabolites (*n* = 45) that have been previously characterized using DTIMS and TWIMS instruments [12,22]. The representative metabolites were analyzed individually over three days and calculated as the percentage of relative standard deviation (%RSD). For accuracy determination, the differences between the ^TW^CCS_N2_ values derived from this study and CCS values previously reported using DTIMS or TWIMS were calculated as a CCS difference error (ΔCCS%) (Supplementary data) [15]. The influence of matrix effects on the CCS values was investigated by spiking human urine (900 μL) with reference standards representing a variety molecular class (*n* = 62) at a concentration of 50 μM (analyzed in ESI^−^ mode). The obtained CCS values of the metabolites in human urine were then compared to those in neat solvent.

### 2.5 Data processing and analysis

Mass spectra and mobility spectra were processed using MassLynx V4.1 and DriftScope V2.9 (Waters), respectively. Metabolite identifications were performed using Progenesis QI MetaScope (Nonlinear Dynamics, Newcastle, UK), searching against the Human Metabolome Database (HMDB) structural database version 5.0 [38]. The search criteria used were as follows; precursor and product ion mass error tolerances ± 20 ppm, isotope similarity ≥ 80%, RT tolerance ± 0.3 min, and ΔCCS ± 2% or ±4 Å^2^ to balance filter efficiency and avoid over-filtering. The HDMS^E^ RAW data files of human urine analyzed in ESI^+^ and ESI^−^ were deposited in the Mass Spectrometry Interactive Virtual Environment (MassIVE) under the accession number MSV000090370, which was implemented at Siriraj Metabolomics Data Warehouse (SiMD) (http://www.metsysbio.com/simd/).

## 3 Results

### 3.1 TWIMS parameters optimization to improve the transmission of VLMs

**Fig. 1:**
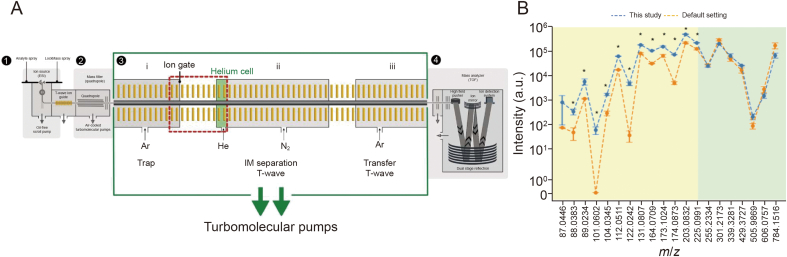
Optimization of traveling wave ion mobility spectrometry (TWIMS) parameters for detecting very-low molecular weight metabolites (VLMs). (A) A schematic presentation of Synapt G2-Si traveling wave ion mobility spectrometry (TWIMS)-quadrupole-time-of-flight (QTOF)-mass spectrometry (MS) used in this study. The instrument consists of four main regions: (1) ion source (electrospray ionization (ESI)), (2) ion selection (quadrupole mass analyzer), (3) IM separation (Tri-Wave region), and (4) mass analysis (time-of-flight mass analyzer). The Tri-Wave region consists of i) a trap cell, ii) TWIMS cell, and iii) an ion transfer cell. The helium cell (green), located at the entrance to the IM region, provides collisional cooling for the gated ion packets as they are accelerated through the pressure gradient entering the IM separation region. The potential gradient between the trap cell and helium cell (region indicated by red dashes) was tuned to minimize dissociation of the labile compounds, whilst maintaining ion transmission across the mass range in this study. In the IMS cell, a low wave height (WH) and high wave velocity (WV) were also optimized to minimize ion dissociation and maintain reasonable IM separation of the studied compounds. (B) A plot of precursor ion intensities of the selected metabolites measured after optimization (blue line) compared to those measured using the initial settings (orange line). ^∗^*P* < 0.05 (*t*-test). *m*/*z*: mass-to-charge ratio.

To improve the transmission of VLM ions, crucial T-wave parameters, including bias voltages, traveling WH, and WV, were systematically tuned and optimized from their original instrument settings (Fig. 1A). A total of 13 reference standards were used as a representative set of VLMs, with *m*/*z* ranging from 87.0446 to 225.0991. In addition, seven reference standards covering *m*/*z* ranging from 255.2334 to 784.1516 were also considered as representative of molecules larger than the VLMs (Table S2). Optimization was performed in ESI^−^ mode based on triplicate measurements of the [M−H]^−^ of selected precursor ions because this mode provided good sensitivity with low background noise. After tuning to maximize the intensities of the representative analyte ions, new optimized values were trap direct current (DC) bias 35 V (default: 45 V), helium cell DC bias 30 V (default: 50 V), along with WH of 30 V and WV 1000 m/s (default: WH = 40 V and WV = 650 m/s), as these conditions provided better sensitivities (*P* < 0.05), when compared to the default instrument parameters, for ions with *m*/*z* < 250, while maintaining an insignificant difference (*P* > 0.05) in the sensitivity for ions with *m*/*z* greater than 250 (Fig. 1B). Considering the ion intensities in the set of representative standards, the ion intensity at *m*/*z* 101.0602 (isovaleric acid) was not detected using default parameters. This could probably be due to ion heating-induced degradation of isovaleric acid during IM separation. However, when the parameters were optimized, the sensitivity of isovaleric acid increased 10 times better than the default settings, allowing to measure the CCS value of isovaleric acid, to our knowledge, for the first time. Under these new conditions, ion heating was alleviated, minimizing changes in gas-phase conformations and increasing the survival rate of the analyte ions, thereby improving the sensitivity of VLMs using TWIMS.

### 3.2 CCS measurement of 174 metabolites

**Fig. 2:**
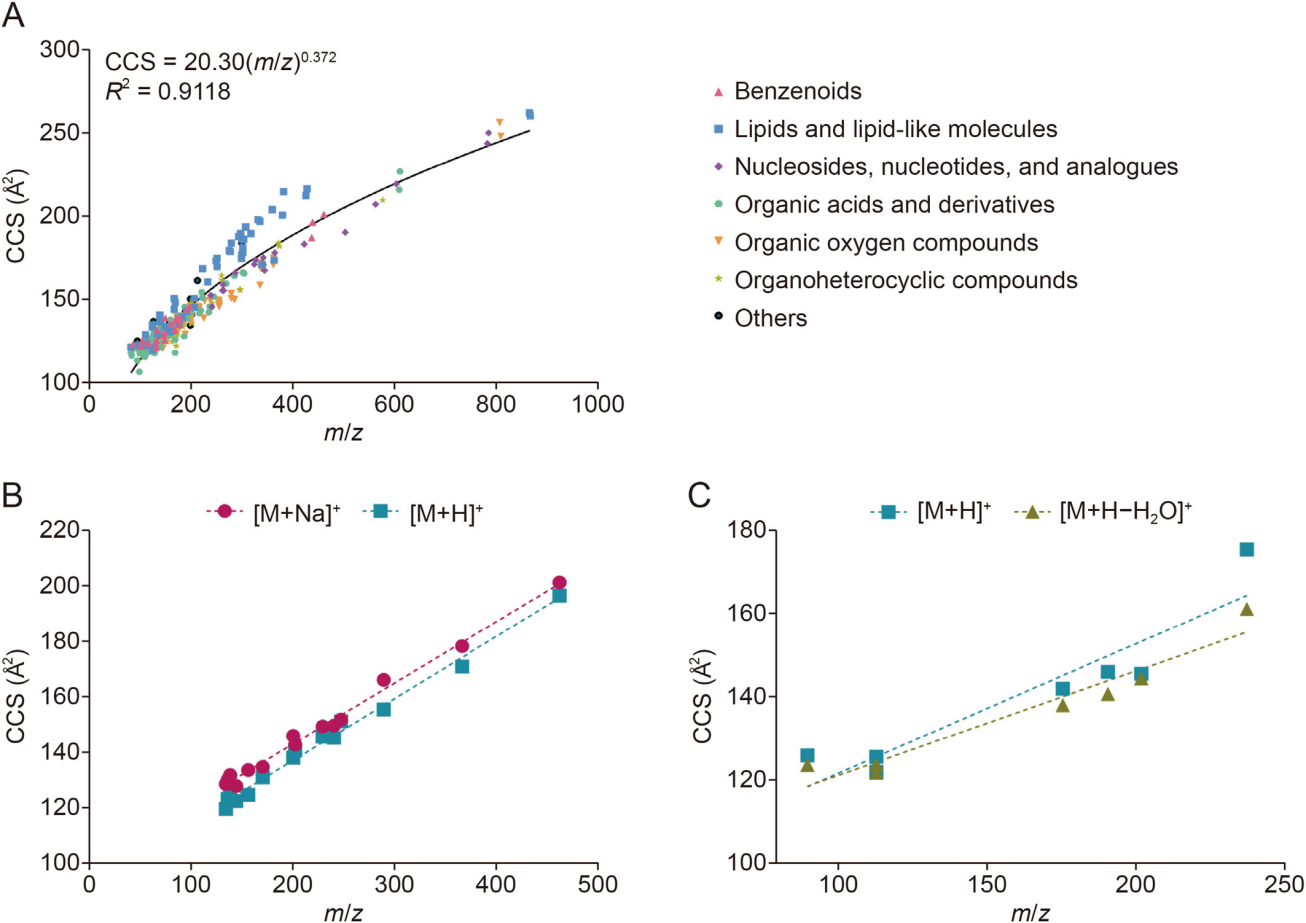
Conformational space plot of mass-to-charge ratio (*m*/*z*) and collision cross-sections (CCS) of the 174 metabolites. (A) The overview distribution of *m*/*z* and CCS for 309 ions categorized into chemical super classes. The black line indicates the main trendline of metabolites. (B) Relationship between *m*/*z* and CCS values of sodiated [M+Na]^+^ and protonated [M+H]^+^ ions. (C) Relationship between *m*/*z* and CCS values of protonated with water loss [M+H–H_2_O]^+^ and protonated [M+H]^+^ ions.

A HILIC UPLC method was implemented to separate the 174 target metabolites within 20 min, with 131 and 154 peaks resolved in ESI^+^ and ESI^−^ modes, respectively. CCS measurements of the 174 standard metabolites were performed in both ESI^+^ and ESI^−^ modes. Thus, 309 ^TW^CCS_N2_ values were obtained in this study (153 for ESI^+^ and 156 for ESI^−^). Fig. 2A shows a plot of *m*/*z* and CCS density distributions acquired from all standard metabolites in both ESI^+^ and ESI^−^ modes. The collection covered an *m*/*z* range from 87.0446 to 868.1255 corresponding to CCS values between 107.41 and 262.11 Å^2^ (Table S3), representing structurally diverse groups of compounds. Based on the experimental TWIMS-derived CCS values from the AllCCS [23] and CCSbase [24] databases, a total of 263 ^TW^CCS_N2_ values were reported for the first time in this study (107 for ESI^+^ and 156 for ESI^−^ modes). A total of 153 ^TW^CCS_N2_ values were identified in ESI^+^, including 68 [M+H]^+^, 64 [M+Na]^+^, 19 [M+H–H_2_O]^+^, and 2 [M]^+^ ions. [M + Na]^+^ ions had higher CCS values, whereas most of the [M+H–H_2_O]^+^ ions had lower CCS values when compared to their respective [M+H]^+^ ions. In general, the CCS values of the [M+Na]^+^ ions increased by 5.85 ± 2.82 Å^2^ (mean ± standard deviation (SD)) relative to those of the [M+H]^+^ ions (Fig. 2B). The larger CCS values of sodium adducts are caused by the conformation of the ion with the charge carrier and the interaction potential of the ion with the drift gas. This is because the atomic radius of sodium ions is much larger than that of protons. In accordance with expectations, the CCS values of [M+H–H_2_O]^+^ ions decreased by 4.11 ± 4.73 Å^2^ (mean ± SD) relative to [M+H]^+^ (Fig. 2C), which may be due to the reduced surface area resulting from the loss of the water molecule. However, in some cases for thymine and uracil, [M+H–H_2_O]^+^ appeared to show larger CCS values than their related [M+H]^+^. The loss of water in these molecules probably tends to change its original cyclic structure, resulting in [M+HH_2_O]^+^ being larger than [M+H]^+^. For ESI^−^ analysis, a total of 156 ^TW^CCS_N2_ values were obtained from 151 [M−H]^−^ and 5 [M+Na–2H]^−^ ions. On average, the CCS values of [M+Na–2H]^−^ ions were 4.72 ± 0.89 Å^2^ (mean ± SD) larger than the CCS values of their corresponding [M−H]^−^ ions. Since small molecules can exhibit multiple adduct states, especially in complex matrices such as urine, this characteristic contributes to difference in their mobilities and CCS values. Therefore, it is crucial to include CCS values of adducts when constructing a CCS library. This inclusion facilitates the differentiation and identification of metabolites during the target screening process, even under varying experimental conditions. A summary of the multidimensional information of the 174 metabolites analyzed under ESI^+^ and ESI^−^ modes is shown in Table S3.

Previous studies have demonstrated a correlation between *m*/*z* and CCS values of different compound classes. These correlation trendlines may delineate compounds into specific chemical super classes [39,40]. In this study, the studied metabolites were classified into seven groups based on ClassyFire [41]. These included benzenoids (*n* = 11), lipids and lipid-like molecules (*n* = 35), nucleosides, nucleotides, and analogs (*n* = 11), organic acids and derivatives (*n* = 52), organic oxygen compounds (*n* = 33), organoheterocyclic compounds (*n* = 23), and others (homogeneous non-metal compounds, organic nitrogen compounds, phenylpropanoids and polyketides, and uncategorized) (*n* = 9). All ion residuals were combined and analyzed both parameters using a power regression model. The all-ion residual trendline (the main trendline) was observed with an *R*^2^ of 0.9118 (Fig. 2A). Additionally, strong correlations between *m*/*z* and CCS of benzenoids (*R*^2^ = 0.9475), lipids and lipid-like molecules (*R*^2^ = 0.9417), nucleosides, nucleotides, and analogs (*R*^2^ = 0.9767), organic acids and derivatives (*R*^2^ = 0.9171), organic oxygen compounds (*R*^2^ = 0.9643), and organoheterocyclic compounds (*R*^2^ = 0.9328) were found (Fig. S1). The ion residuals of each compound super class were then plotted along the main trend line to demonstrate the conformational space occupied by ion residuals in each super class. The trendlines for almost the entire metabolite super class seemed to be distributed relatively near the main trendline, implying that the mass of the small molecules influenced the CCS. On the contrary, lipids and lipid-like molecules were well differentiated and deviated above the main trend line (*m*/*z* ≈ 200−400) (Fig. S1). The lipid super class used in this study was divided into seven subclasses, including fatty acids and conjugates, fatty acid esters, linoleic acid and derivatives, vitamin D and derivatives, quinone and hydroquinone lipids, fatty acyl glycosides, and fatty acyl thioesters, reflecting a wide range of structural diversity. In addition, lipids tend to have a lower gas-phase density, resulting in a larger CCS compared to other biomolecules of similar mass [40]. A non-linear correlation between *m*/*z* and CCS values has been reported for lipids, which is consistent with the diverse molecular shapes and elemental compositions within subclasses that can be well differentiated in conformational space analysis [10].

The rate at which ions travel through the IM chamber is significantly influenced by their interactions with the drift gas. Smaller ions present a smaller area and therefore undergo fewer collisions, and experience less “resistance” moving through the TWIMS device compared to larger ions. While this explains the general correlation between *m*/*z* and CCS values within compound classes [42], *m*/*z* alone is not sufficient for CCS prediction, as CCS depends not only on the three-dimensional (3D) conformation of the molecule, but also on the location of the charge state, such as the protomer. As gas-phase analyte ions continually move rotationally and translationally during TWIMS separation, isomers with branched and extended structures experience greater collisions with the buffer gas than those that are structurally compact, which leads to the separation of isomeric compounds. For example, l-leucine and l-isoleucine are difficult to distinguish without comparing their retention times. However, the extracted ion mobiligrams of their [M+H]^+^ ions clearly show a different arrival time distribution, in which l-leucine (CCS = 131.44 Å^2^) showed a faster drift time of 0.87 ms compared to that of l-isoleucine (CCS = 133.13 Å^2^) of 0.92 ms (Fig. S2A). The IMS used in this study was not enough to resolve them in the mixture (Fig. S2B). A previous study has shown that a resolving power of IMS at least 118 Ω/ΔΩ was predicted to adequately separate these constitutional isomers [43].

### 3.3 Experimental ^TW^CCS_N2_ values are accurate, highly repeatable, and matrix independent

**Fig. 3:**
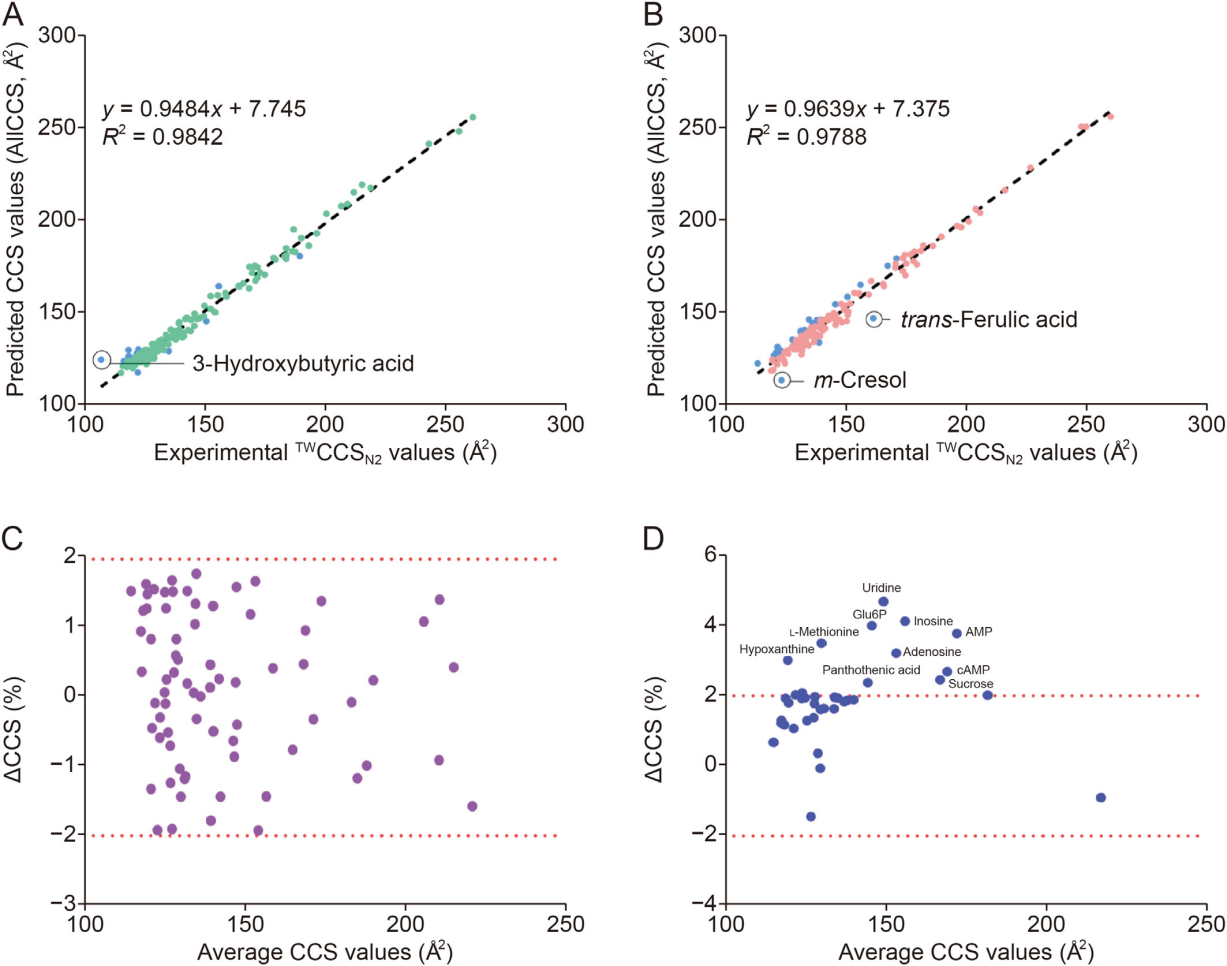
Correlation and deviation analysis of collision cross-sections (CCS) measurement. (A, B) Correlation between traveling wave ion mobility spectrometry (TWIMS)-derived CCS values (^TW^CCS_N2_ values) for all the studied ions and predicted CCS values from computational machine learning (AllCCS) in negative electrospray ionization (ESI^−^) (A) and positive electrospray ionization (ESI^+^) (B) modes. (C, D) Percentage relative plots display ΔCCS deviations between the measured ^TW^CCS_N2_ values in this study and the literature drift tube ion mobility spectrometry (DTIMS)-derived CCS values (C) and TWIMS-derived CCS values (D). Glu6P: glucose-6-phosphate; AMP: adenosine monophosphate; cAMP: cyclic AMP.

The precision of experimental ^TW^CCS_N2_, measured during consecutive inter-day (*n* = 3) experiments using a set of metabolites (*n* = 45) for which both DTIMS- and TWIMS-derived CCS values have been previously reported [12,22], was evaluated. The inter-day experiments showed a high repeatability under the new measurement settings, with a %RSD of CCS values < 0.85% (Table S4). The experimental ^TW^CCS_N2_ values were further compared to the predicted CCS values from the online machine learning database AllCCS [23]. In general, only 54% of the experimental ^TW^CCS_N2_ measurements in both ESI^+^ and ESI^−^ modes yielded ΔCCS of ±2% when compared with the predicted CCS values (Table S5). The highest CCS deviation of 14% was observed for deprotonated 3-hydroxybutyric acid, for which the predicted CCS value was much higher than the experimental CCS value (Fig. 3A). In some cases, the experimental ^TW^CCS_N2_ values yielded large CCS deviations, for example, water loss ion of *m*-cresol (ΔCCS = 8%) and sodium adduct of *trans*-ferulic acid (ΔCCS = 9%) (Fig. 3B). Although a strong correlation between experimental ^TW^CCS_N2_ values and predicted CCS values has been observed in ESI^+^ (*R*^2^ = 0.9768) and ESI^−^ (*R*^2^ = 0.9842), with some exceptions, 46% of studied ions have CCS deviations of more than ±2%. Several factors could cause deviations in predicted CCS values calculated using machine learning models from the experimental values, such as the structural diversity of the charge isomers derived from different experiments, the quality of the training data, and the prediction model [2,44]. These factors may lead to uncertainty for CCS prediction. This highlights the necessity for comprehensive experimental CCS measurements covering the whole range of biomolecules.

To evaluate the accuracy of CCS measurement in VLMs measured under new parameters, experimental ^TW^CCS_N2_ values measured in this study were compared to the experimental CCS values that have been previously published using either DTIMS or TWIMS [12,22]. A total of 73 and 38 target metabolites had previously reported DTIMS and TWIMS CCS values, respectively, and only 54 and 30 of these metabolites were VLMs (Table S6). The difference between experimental ^TW^CCS_N2_ values measured in this study and the literature DTIMS-derived CCS value was expressed as Δ^DT^_lit_^/TW^CCS_N2_%, and the difference between experimental ^TW^CCS_N2_ values measured in this study and the literature TWIMS-derived CCS value was expressed as Δ^TW^_lit_^/TW^CCS_N2_%. All Δ^DT^_lit_^/TW^CCS_N2_% values were small (±1.9%) (Fig. 3C), while 74% of the Δ^TW^_lit_^/TW^CCS_N2_% was within ±2%. (Fig. 3D). For VLMs, more than 90% of the Δ^DT^_lit_^/TW^CCS_N2_% and Δ^TW^_lit_^/TW^CCS_N2_% values were within the range of ±2% error. The greatest Δ^TW^_lit_^/TW^CCS_N2_% (>±2%) was observed for 10 compounds, in which the highest deviation was attributed to the deprotonated uridine ion (Δ^TW^_lit_^/TW^CCS_N2_% = 4.7%) (Fig. 3D and Table S6). Because the DTIMS derived of CCS values is based on the Mason-Schamp equation [45], CCS values with a high degree of precision and accuracy are generally warranted [14,46]. TWIMS-derived CCS values, on the other hand, are indirectly determined by CCS calibration based on a small number of chemicals with known DTIMS-derived CCS values. When calibrating the drift times to a CCS calibration curve, it was assumed that this was independent of the calibrant. However, the choice of CCS calibrant affects the accuracy of measurements, as structurally dissimilar classes of compounds have been shown to lead to larger errors in calibrated CCS values than structurally matched calibrants [47]. Moreover, a recent study has shown that systematic errors were observed in CCS measurements, and the non-uniform arrival time distributions of some calibrant ions have been found in the Major Mix IMS/TOF analyzed on the DTIMS instrument. These factors may affect the reliability of CCS calibration, especially in a high-resolution IMS [44].

There is currently no consensus over which calibration standard is optimal for the TWIMS setup for VLMs. Generally, polyalanine and Major Mix IMS/TOF calibrant (also composed of polyalanine) are the most widely used for TWIMS CCS calibration [48]. It should be noted that this study used the Major Mix IMS/TOF calibration, which differed from previous reports that used only polyalanine [12,34]. As such, this could partially explain the small deviations from the CCS values in the literature. Nonetheless, this study demonstrated that CCS values for VLMs are generally in agreement with the experimental CCS values reported using different instrumental platforms and conditions. The results supported that optimized method for CCS determination has no impact on the accuracy and reliability of CCS values for the studied metabolites that are comparable to the standard measurements. To use the CCS value as an additional molecular descriptor, the CCS values of the target metabolites were determined using a biological matrix. This investigation was performed on a pooled human urine sample fortified with a mixture of selected reference standards (*n* = 62) (analyzed in ESI^−^ mode). The CCS values of the target metabolite measured in the human urine matrix deviated slightly from those measured in the neat solution, with ΔCCS error within ±1.92% (Table S7). This result indicates that CCS measurement of metabolites is independent of matrix effects. Based on this result, ΔCCS of ±2% was applied during the screening of metabolites in the urine samples.

### 3.4 Four-dimensional library enhances the accuracy of metabolite identification

**Fig. 4:**
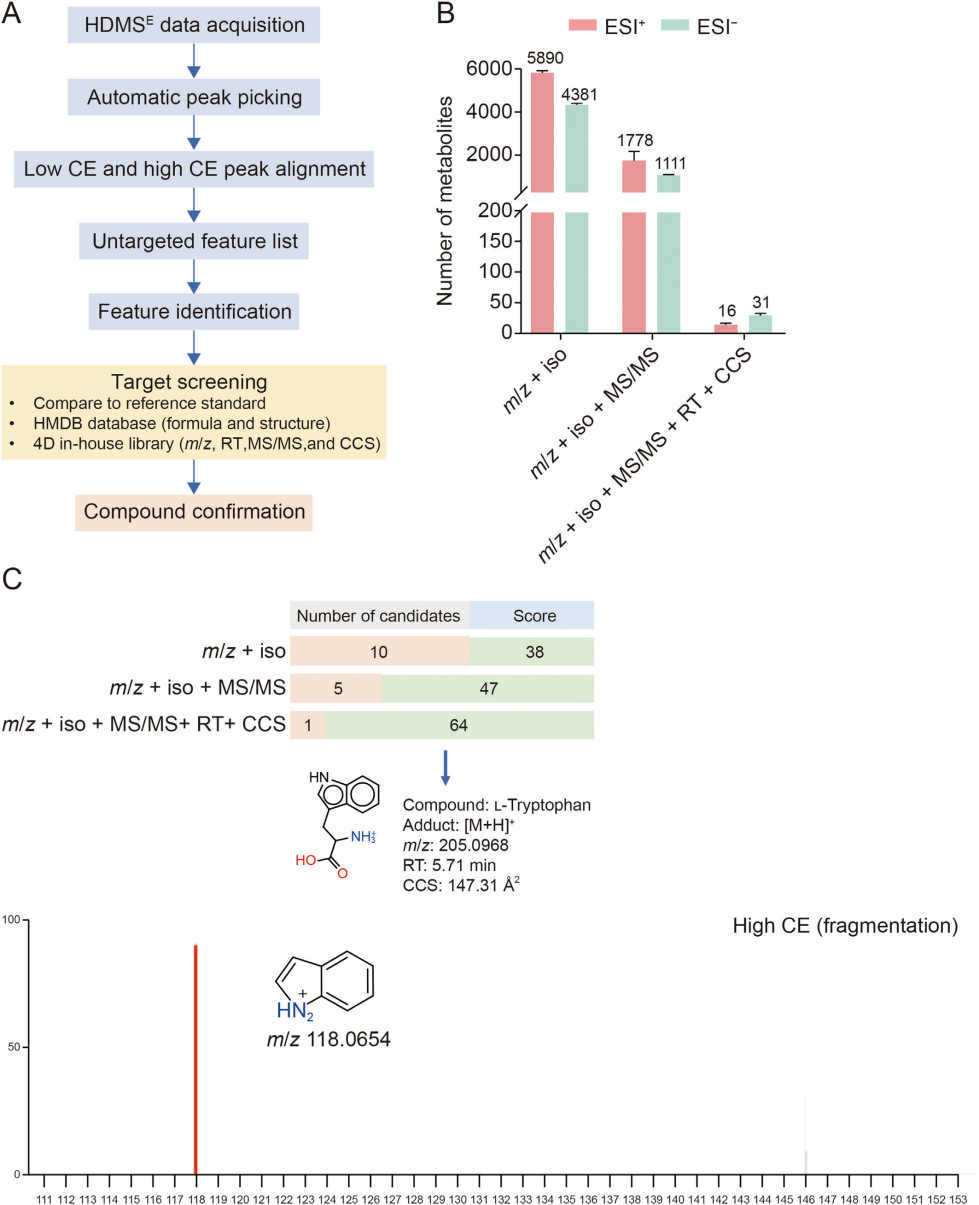
An overview of the workflow for the proposed ultra-performance liquid chromatography (UPLC)/traveling wave ion mobility spectrometry (TWIMS)-quadrupole-time-of-flight (QTOF)-mass spectrometry (MS) analysis for target screening and compound identification. (A) High definition MS^E^ (HDMS^E^) data processing for screening target metabolites using Progenesis QI. (B) Average numbers of metabolite candidates given by different matching criteria. (C) Example of identification of l-tryptophan in human urine using measured mass-to-charge ratio (*m*/*z*), isotopic similarity (iso), retention time (RT), fragment ions (MS/MS), and collision cross-sections (CCS) value. CE: collision energy; 4D: four-dimensional; ESI: electrospray ionization.

To facilitate metabolite identification, a 4D library of 174 metabolites containing molecular descriptors, including *m*/*z*, RT, MS/MS spectra, and CCS values was generated (Fig. 4A). Subsequently, pooled human urine samples with triplicate injections were analyzed, and the library was applied to search for target analytes. On average, 5,890 ± 106 (mean ± SD) and 4,381 ± 69 (mean ± SD) metabolite candidates were identified using the traditional approach (*m*/*z* and isotope similarity) in ESI^+^ and ESI^−^ modes, respectively. Although large numbers of candidate metabolites are “identified”, many of these are isobaric or isomeric compounds, resulting in a large number of possible candidates for a single detected feature. Because isomers have the same elemental composition, the isotope distribution will be identical, and isobaric compounds often have similar compositions because organic compounds are predominantly composed of C, H, N, and O, resulting in very similar isotopic distributions. This highlights an important drawback of the traditional approach for metabolite identification that relies only on mass accuracy and isotope similarity. However, by including additional parameters such as RT, MS/MS spectra, and CCS, the number of candidates that require further validation is reduced from an average of 5890 to 16 metabolites in ESI^+^ and from 4381 to 31 metabolites in ESI^−^ (Fig. 4B). For example, a feature, extracted from HDMS^E^ data in ESI^+^ mode which had RT of 5.71 min and *m*/*z* of 205.0968, was subjected to metabolite identification using the HMDB database with the matching criteria as follows: mass error ± 20 ppm and isotopic similarity ≥ 80%, and RT tolerance ± 0.3 min. The approach yielded 10 metabolite candidates with similar identification scores of 38; therefore, no candidate could be assigned. When introducing MS/MS fragment matching criteria using either fragment ion libraries created using reference standards (Table S8) or theoretical *in silico* fragments (mass error, ±20 ppm) to the previous metabolite identification workflow, five candidates were removed. The remaining five candidates with the highest identification scores (score = 47) were glycyl-phenylalanine, phenylalanyl-glycine, l-tryptophan, d-tryptophan, and (±)-tryptophan. After adding the CCS matching criteria (ΔCCS ± 2%), l-tryptophan had the highest identification score (score = 64) (Fig. 4C). The presence of l-tryptophan was confirmed by spiking the l-tryptophan standard (50 μM, 5 μL injected (0.25 nmol)) in human urine samples and analyzed them in the ESI^+^ mode. The results showed that all matching criteria (*m*/*z*, RT, MS/MS, and CCS) were within the given tolerance.

In the ESI^+^ and ESI^−^ modes, 23 and 44 metabolites from various chemical super classes were identified in human urine samples, respectively. All identified metabolites were assigned as level 1 identification confidence according to the Metabolomic Standard Initiative [49], because the assignments were made against three independent orthogonal properties from the reference standards. Details of the 55 identified metabolites in human urine, along with their molecular descriptors, including *m*/*z*, adducts, molecular formula, identification score, fragmentation score, and isotope similarity, as well as their chemical characterization categorized based on super classes are provided in Table 1 and Fig. S3, respectively. The total ion chromatogram (TIC) of human urine, and the extract ion chromatogram (XIC) of the metabolites analyzed in ESI^+^ and ESI^−^ modes are shown in Figs. S4−S6, respectively.

**Table 1:** Metabolite identification in human urine under positive electrospray ionization (ESI^+^) and negative electrospray ionization (ESI^−^) modes analyzed by Progenesis QI software.

No.	Compounds	HMDB ID	Monoisotopic mass (Da)	Adduct	*m*/*z*	RT (min)	CCS (Å^2^)	Score	MS/MS score	Δppm	ΔRT (min)	ΔCCS (Å^2^)	Ιsotope similarity
1	(*S*)-2-Methylbutanoic acid	HMDB33742	102.0680	[M−H]^−^	101.0591	1.21	123.36	52.5	0	−16.35	−0.29	−0.12	97.68
2	Isovaleric acid	HMDB00718	102.0680	[M+Na–2H]^−^	123.0447	0.64	122.50	64.5	0	19.03	0.26	0	96.47
3	2-Hydroxybutyric acid	HMDB00008	104.0473	[M−H]^−^	103.0382	2.15	119.87	52.4	0	−18.35	−0.20	−0.11	95.03
4	*m*-Cresol	HMDB02048	108.0575	[M−H]^−^ [M+H]^+^	107.0504 109.0655	0.70 0.97	122.60 126.19	74.7 66.8	91.3 58.9	1.59 6.70	0.05 −0.20	−0.13 0	98.60 81.40
5	Uracil	HMDB00300	112.0272	[M−H]^−^	111.0186	1.10	114.01	44.2	0	−12.08	−0.02	−1.76	94.61
6	Creatinine	HMDB00562	113.0589	[M+H]^+^	114.0667	2.81	127.17	48.9	0	4.80	0.02	3.59	99.41
7	*α*-Ketoisovaleric acid	HMDB00019	116.0473	[M−H]^−^	115.0384	2.08	121.72	44.6	0	−14.17	0.08	−1.70	94.02
8	Caproic acid	HMDB00535	116.0837	[M−H]^−^	115.0748	1.08	127.92	41.6	0	−14.28	−0.18	−1.67	96.78
9	Succinic acid	HMDB00254	118.0266	[M−H]^−^	117.0174	1.58	116.71	41.4	0	−16.49	−0.08	−1.73	94.83
10	2-Methyl-3-hydroxybutyric acid	HMDB00354	118.0629	[M−H]^−^	117.0539	1.20	124.64	52.2	0	−15.63	−0.10	−0.14	93.99
11	2-Hydroxy-2-methylbutyric acid	HMDB01987	118.0629	[M−H]^−^	117.0544	1.78	123.08	42.5	0	−10.98	−0.23	−1.70	98.46
12	Betaine	HMDB00043	117.0793	[M+H]^+^	118.0877	7.14	128.28	62.0	81.9	2.77	−0.02	2.95	94.92
13	2,4-Dihydroxybutanoic acid	HMDB00360	120.0422	[M−H]^−^	119.0338	4.96	119.73	59.2	0	−9.55	−0.05	−0.12	98.47
14	Nicotinic acid	HMDB01488	123.0320	[M−H]^−^	122.0243	2.66	119.45	44.8	0	−3.35	0.08	−1.71	92.93
15	Taurine	HMDB00251	125.0146	[M−H]^−^	124.0072	6.29	119.27	75.4	84.8	−1.79	−0.15	−0.12	99.33
16	Thymine	HMDB00262	126.0429	[M−H]^−^	125.0339	1.21	119.18	41.7	0	−13.85	−0.18	−1.71	98.48
17	Pyroglutamic acid	HMDB00267	129.0425	[M+H]^+^	130.0501	6.47	127.14	44.2	0	15.16	−0.30	1.12	96.83
18	3-Methyl-2-oxopentanoic acid	HMDB00491	130.0629	[M−H]^−^	129.0540	2.00	126.56	53.9	0	−13.22	−0.07	−0.15	95.81
19	Ketoleucine	HMDB00695	130.0629	[M−H]^−^	129.0541	1.86	129.55	43.6	0	−12.20	0.07	1.34	93.00
20	2-Methylhexanoic acid	HMDB31594	130.0993	[M−H]^−^	129.0898	1.03	133.93	50.3	0	−17.45	−0.23	−0.18	92.18
21	4-Hydroxyproline	HMDB00725	131.0582	[M−H]^−^	130.0496	7.36	123.43	42.1	0	−10.51	0.19	−3.19	93.67
22	l-Isoleucine	HMDB00172	131.0946	[M−H]^−^ [M+H]^+^	130.0852 132.0993	6.95 6.47	129.46 131.50	41.9 65.2	0 0	−16.79 −19.86	−0.27 0.00	−1.64 1.63	92.85 92.85
23	l-Leucine	HMDB00687	131.0946	[M−H]^−^ [M+H]^+^	130.0861 132.1007	6.31 6.29	130.93 131.50	54.6 48.6	0 0	−9.37 −9.22	0.15 −0.03	−0.17 −0.06	92.84 92.84
24	Methylsuccinic acid	HMDB01844	132.0422	[M−H]^−^	131.0329	1.40	121.81	53.3	0	−9.12	−0.12	−0.13	98.86
25	Hypoxanthine	HMDB00157	136.0385	[M−H]^−^ [M+H]^+^	135.0314 137.0478	2.39 2.36	119.94 128.07	62.1 56.8	84.9 77.1	1.47 15.03	−0.08 −0.08	−1.68 3.02	94.49 97.44
26	2-Aminobenzoic acid	HMDB01123	137.0476	[M−H]^−^	136.0389	1.00	124.47	42.9	0	−10.76	−0.03	0.42	91.92
27	4-Hydroxybenzoic acid	HMDB00500	138.0316	[M+H–H_2_O]^+^ [M−H]^−^	121.0290 137.0242	0.72 0.90	124.91 119.94	61.1 44.2	0 0	4.01 −1.60	−0.01 −0.23	0 −1.67	92.06 98.71
28	Methyl heptanoate	HMDB31478	144.1150	[M−H]^−^	143.1051	1.03	138.40	43.2	7.2	−18.65	0.10	1.20	91.18
29	l-Glutamic acid	HMDB00148	147.0531	[M−H]^−^	146.0461	8.16	125.24	61.6	0	1.53	0.05	−0.15	93.46
30	Guanine	HMDB00132	151.0494	[M−H]^−^	150.0431	3.72	123.47	45.5	0	6.10	−0.07	−1.65	92.78
31	4-Hydroxyphenyl acetate	HMDB00020	152.0473	[M−H]^−^	151.0407	0.64	127.85	44.6	0	4.07	0.03	−1.62	95.68
32	D-Arabitol	HMDB00568	152.0684	[M−H]^−^	151.0595	3.31	126.39	54.1	0	−11.07	−0.15	−0.15	97.29
33	3-Methyladipic acid	HMDB00555	160.0735	[M−H]^−^	159.0657	1.08	131.66	54.6	0	−3.78	−0.15	−0.18	99.33
34	l-Phenylalanine	HMDB00159	165.0789	[M−H]^−^ [M+H]^+^	164.0698 166.0871	6.00 5.94	138.32 139.35	64.0 52.8	0 0	−11.89 5.16	0.02 −0.02	−0.19 2.21	96.81 95.94
35	Uric acid	HMDB00289	168.0283	[M+H]^+^	169.0356	3.19	133.36	60.5	0	−0.27	−0.17	0	98.71
36	l-Arginine	HMDB00517	174.1116	[M+H]^+^	175.1185	10.71	138.83	52.9	24.5	−2.63	−0.10	2.20	91.83
37	Ascorbic acid	HMDB00044	176.0320	[M+H]^+^	177.0400	0.72	134.39	73.9	34.8	3.43	0	0	90.48
38	Hippuric acid	HMDB00714	179.0582	[M+H]^+^	180.0656	2.16	138.57	60.5	0	0.26	0	1.07	90.75
39	l-Tyrosine	HMDB00158	181.0738	[M−H]^−^ [M+H]^+^	180.0649 182.0818	6.77 6.59	141.44 142.76	64.1 48.3	0 0	−9.73 3.61	0.02 0.08	−0.21 1.67	99.45 95.70
40	3-(3-Hydroxyphenyl)-3-hydroxypropanoic acid	HMDB02643	182.0579	[M−H]^−^	181.0496	1.17	138.72	68.3	0	−5.72	0	−0.20	98.18
41	3-Hydroxyphenyllactate	HMDB29232	182.0579	[M−H]^−^	181.0497	2.24	140.06	66.2	0	−4.97	0	−0.20	89.87
42	Sorbitol	HMDB00247	182.0790	[M−H]^−^	181.0693	4.58	133.26	60.4	0	−13.78	0.02	−0.18	92.32
43	Galactitol	HMDB00107	182.0790	[M−H]^−^	181.0696	5.11	133.26	53.4	0	−12.09	−0.27	−0.18	98.48
44	Azelaic acid	HMDB00784	188.1048	[M−H]^−^	187.0955	0.97	138.42	61.6	92.7	−10.56	−0.17	−1.54	97.70
45	Kynurenic acid	HMDB00715	189.0425	[M−H]^−^ [M+H]^+^	188.0340 190.0507	4.10 4.10	134.30 138.07	59.8 60.4	0 72.9	−7.14 4.58	0.03 −0.09	−0.19 2.19	95.91 98.71
46	Phenylacetylglycine	HMDB00821	193.0738	[M−H]^−^	192.0655	2.36	148.61	43.1	0	−5.86	−0.20	2.33	97.42
47	Glucuronic acid	HMDB00127	194.0426	[M+H–H_2_O]^+^	177.0400	0.72	134.39	81.8	73.9	3.11	0	0	90.30
48	l-Tryptophan	HMDB00929	204.0898	[M−H]^−^ [M+H]^+^	203.0824 205.0968	5.74 5.71	148.10 147.31	76.0 64.0	95.1 44.1	−0.96 −1.92	0.12 −0.06	−0.23 −0.45	97.08 94.12
49	Xanthurenic acid	HMDB00881	205.0375	[M−H]^−^ [M+H]^+^	204.0265 206.0458	4.60 4.47	134.98 138.81	46.6 76.4	0 91.0	−18.15 4.82	0.05 0	−1.54 1.99	97.75 92.50
50	l-Kynurenine	HMDB00684	208.0847	[M+H]^+^	209.0958	5.71	144.92	50.7	0	17.76	0.03	1.07	94.51
51	Pantothenic acid	HMDB00210	219.1106	[M−H]^−^ [M+H]^+^	218.1033 220.1187	2.19 1.98	147.48 148.10	63.5 52.9	84.6 18.8	−0.24 −0.80	−0.11 −0.02	1.04 2.47	89.56 98.45
52	Carnosine	HMDB00033	226.1065	[M+H]^+^	227.1141	11.76	149.23	52.4	0	0.95	−0.04	−2.42	88.74
53	Biotin	HMDB00030	244.0881	[M−H]^−^ [M+H]^+^	243.0809 245.1002	1.75 1.81	155.19 148.62	46.2 58.6	0 6.7	0.18 19.55	0.03 −0.07	2.16 0	91.19 95.66
54	Adenosine	HMDB00050	267.0967	[M+H]^+^	268.1044	2.39	153.43	52.3	40.9	1.32	−0.15	−2.46	95.70
55	Inosine	HMDB00195	268.0807	[M−H]^−^	267.0713	3.12	156.85	42.5	0	−8.09	0.28	−2.62	96.78

HMDB: Human Metabolome Database; *m*/*z*: mass-to-charge ratio; RT: retention time; CCS: collision-cross section; MS/MS: tandem mass spectrometry.

## 4 Discussion

In this study, the successful optimization of an analytical method using UPLC-TWIMS-QTOF-MS to enhance ion transmission for measuring CCS values in VLMs was described. The use of CCS values in combination with *m*/*z*, adducts, RT, and MS/MS to facilitate metabolite identification in human urine was also validated and demonstrated. TWIMS-MS has long been known for its poor transmission of very labile compounds due to ion heating and dissociation before reaching the MS analyzer [29,32,33,36]. This has a direct impact on the ability to make reliable CCS measurements of VLMs; with only 6% of VLMs having CCS values previously reported [27]. In this study, a total of 309 ^TW^CCS_N2_ values were measured from 174 reference standards. Of these, 263 ^TW^CCS_N2_ values were reported for the first time, and more than 72% of these were CCS values of VLMs. Compared to the original instrument settings, the optimized TWIMS conditions increased the precursor ion intensities for VLMs without compromising the sensitivity of larger molecules (more than 250 Da). This could be due to the low bias voltages between the trap and IM regions that lead to lower injection energies of ions and/or the low WH, which is directly correlated with the electric field intensity applied to the ions inside the IM cell. Alternatively, the high WV used in the optimized method may sweep the small ions through the IMS cell faster, thus increasing the chance of detection by TOF before dissociation. The method not only enhanced the coverage of VLMs but also offered accuracy comparable to standard settings for measuring the CCS values of VLMs. This is supported by the consistency with previously reported CCS values obtained using the TWIMS (same instrument) and standardized DTIMS instruments. Overall, despite minor adjustments to the TWIMS parameters, this optimization enabled the measurement of CCS values for small molecules, allowing for an expanded spectrum for the detection of VLMs in biological sample. With the rapid growth of CCS applications in metabolite identification, the collection of ^TW^CCS_N2_ values in this study will be valuable for the scientific community and clinical metabolomics research.

Metabolite identification is a crucial step in translating data into meaningful biological contexts. According to the Metabolomics Standard Initiative (MSI), currently two orthogonal data, including *m*/*z* and MS/MS matching, are typically used to identify metabolite candidates [49]. However, numerous factors, including low abundances and coelution of metabolites in complex matrices, can impede metabolite identification. This study demonstrates the significance of CCS values as an additional molecular descriptor, in addition to *m*/*z* and MS/MS spectra, to narrow down possible candidate metabolites and enhance confidence in metabolite identification. Based on previous studies of the human urine metabolome, Zhou et al. [20] identified 1,284 putative metabolites using DTIMS-TOF-MS, whereas Di Poto et al. [21] reported 362 putative metabolites using TIMS-TOF-MS. However, almost all annotated metabolites in both studies were not verified by reference standards. In 2014, Paglia et al. [12] confirmed 46 detected metabolites in human urine using TWIMS-QTOF-MS. Using metabolite identification based on the closeness of the match in the in-house library: mass error of ±20 ppm, RT tolerance of ±0.3 min, and CCS deviation of ±2% or ±4 Å^2^, a total of 55 metabolites were assigned as level 1 according to the MSI guideline. Moreover, 96% (53/55) of the identified metabolites were VLMs. The higher number of confirmed metabolites obtained in this study was a result of the addition of ^TW^CCS_N2_ values of VLMs, providing a higher coverage of metabolites in human urine with the highest confidence. However, this study had several limitations. Although the in-house library currently contains 309 ^TW^CCS_N2_ values, many metabolites are not included in this library. This highlights the importance of creating, continuing to expand, and updating experimental CCS values, which can ultimately aid in improving metabolite identification for biological samples. The use of predicted CCS values based on machine learning and deep learning has also been proposed to aid in metabolite annotation [20,23,50]. Although the typical CCS deviations between the predicted and experimental values are within ±3% [23,51], larger CCS deviations were observed in this study. Better predictions will be expected if the CCS prediction tool uses data acquired using the same instrument and calibration for machine learning [52]. An additional limitation is that the current TWIMS system used in this study has a resolution of 40–50 Ω/ΔΩ [3,53]. It may not resolve all the studied structural isomers if their CCS values and RTs are close and within the tolerances. For example, sorbitol and mannitol (in ESI^+^ mode) were not separated using TWIMS because their CCS values and RTs were identical. In addition, l-leucine and l-isoleucine had CCS values that were within 1.2% of each other (from the separate analysis); therefore, making within ±2% CCS tolerance would not allow differentiation between them in the standard mixture. In the latter case, a higher IMS resolving power (>200 Ω/ΔΩ) would provide more efficient separation [3,54,55]. A recent study has shown that the structures for lossless ion manipulation IMS and cyclic IMS are able to separate isomers of biomolecules [[56], [57], [58]], and protomers of antibiotics [59]. These techniques are promising for differentiating structural isomers of metabolites. Beside IMS, chromatography is also important to establish the expected RT order for isomer separation, especially in the absence of standardized CCS values. The challenge of isomer identification using CCS values still exists when aiming for “standards-free” identification.

## 5 Conclusions

In conclusion, a UPLC/TWIMS-QTOF-MS method was developed to improve the measurement of VLMs. A library containing 309 ^TW^CCS_N2_ values from 174 reference standards was constructed. Of these, 263 ^TW^CCS_N2_ values have been reported for the first time. Similar to the standard methods, the ^TW^CCS_N2_ measurements using the new method provided high inter-day precision (RSD < 1%) and were not affected by the complex matrix in human urine (ΔCCS ± 1.92%). Finally, the potential of this analytical method and library for metabolite identification in human urine was demonstrated. The use of CCS values in combination with *m*/*z*, isotopic similarity, RT, and MS/MS matching greatly reduced the number of candidate metabolites requiring validation and improved metabolite identification confidence.

## CRediT author statement

**Alongkorn Kurilung:** Conceptualization, Methodology, Formal analysis, Writing - Original draft preparation; **Suphitcha Limjiasahapong** and **Khwanta Kaewnarin:** Investigation, Formal analysis, Writing - Reviewing and Editing; **Pattipong Wisanpitayakorn**, **Narumol Jariyasopit**, **Kwanjeera Wanichthanarak**, **Sitanan Sartyoungkul**, and **Stephen Choong Chee Wong:** Formal analysis, Writing - Reviewing and Editing; **Nuankanya Sathirapongsasuti**, **Chagriya Kitiyakara**, and **Yongyut Sirivatanauksorn:** Resources, Funding acquisition; **Sakda Khoomrung:** Conceptualization, Methodology, Formal analysis, Writing - Original draft preparation, Funding acquisition, Supervision.

## Declaration of competing interest

The authors declare that there are no conflicts of interest.
