# Supplementary material for: Measurement of very low-molecular weight metabolites by traveling wave ion mobility and its use in human urine samples
Source: J Pharm Anal. 2023 Dec 16;14(5):100921. doi: 10.1016/j.jpha.2023.12.011 (PMC11127212; doi:10.1016/j.jpha.2023.12.011)
Supplement: Multimedia component 1 [file mmc1.docx]

**Supplementary data**

**Measurement of Very Low-Molecular Weight Metabolites by Traveling Wave Ion Mobility and Its Use in Human Urine Samples**

**Table of Contents**

[**Supplementary materials and methods** 3](#_Toc144291072)

[**Supplementary results** 6](#_Toc144291073)

[**Fig. S1.** Correlations between *m/z* and CCS values of the studied compound categorized by super classes 6](#_Toc144291074)

[**Fig. S2.** (A) The overlaid of extracted ion mobiligram at *m/z* 132.1024 ± 5 ppm for [M+H]^+^ ions of structural isomers L-leucine and L-isoleucine. (B) The extracted ion mobiligram of the mixture of L-leucine and L-isoleucine. 7](#_Toc144291075)

[**Fig. S3.** Level 1 metabolite annotation in district chemical super classes identified in human urine. 7](#_Toc144291076)

[**Fig. S4.** Total ion chromatogram (TIC) of human urine analyzed in (A) ESI^+^ and (B) ESI^−^ modes. 8](#_Toc144291077)

[**Fig. S5.** Extract ion chromatogram (XIC) of metabolites identified in human urine analyzed in ESI^+^ mode. 9](#_Toc144291078)

[**Fig. S6.** Extract ion chromatogram (XIC) of metabolites identified in human urine analyzed in ESI^−^ mode. 10](#_Toc144291080)

[**Table S2.** Representative very low-molecular weight metabolites (VLMs) used for optimizing the TWIMS parameters in this study. 12](#_Toc144291083)

[**Table S4.** Inter-day precision (*n* = 3) for 45 representative metabolites. 13](#_Toc144291084)

[**Table S6.** Interlaboratory comparison of CCS values. 15](#_Toc144291086)

[**Table S7.** Comparison of CCS values in our library and metabolites detected in a fortified urine matrix. 18](#_Toc144291089)

# **Supplementary materials and methods**

**Chemicals, reagents, and standards**

Acetonitrile, methanol, formic acid (Fisher Scientific, Geel, Belgium), ammonium formate, (Thermo Fisher Scientific, Ward Hill, USA) and ammonium acetate (Loba Chemie, Mumbai, India) were HPLC grade. Ultra-pure water (18.2 MΩ cm, 25 °C, < 10 ppb) was obtained from the Milli-Q purification system (Millipore, Molsheim, USA). A total of 174 analytical standards from seven super classes of polar metabolites (classified based on the ClassyFire database [41]) were purchased from Sigma-Aldrich (Stuttgart, Germany). These included benzenoids (*n* = 11), lipids and lipid-like molecules (*n* = 35), nucleosides, nucleotides, and analogues (*n* = 11), organic acids and derivatives (*n* = 52), organic oxygen compounds (*n* = 33), organoheterocyclic compounds (*n* = 23), homogeneous non-metal compounds (*n* = 1), organic nitrogen compounds (*n* = 3), phenylpropanoids and polyketides (*n* = 3), and uncategorized (*n* = 2). Individual stock standard solutions were prepared in water, methanol, or dimethyl sulfoxide according to their solubility. Working standard solutions with concentrations ranging from 20−100 µM were used for CCS determination. The Major Mix IMS/TOF Calibration Kit (Waters, Milford, MA, USA) was used as directed. The LockMass and LockCCS solution consisted of 200 pg/µL of leucine enkephalin (Waters, Milford, MA, USA) in acetonitrile:water (50:50, *V*/*V*) with 0.1% formic acid for ESI^+^, and without formic acid for ESI^−^ acquisition.

**Sample and sample preparation**

***Human urine sample***

This study was approved by the Ethical Clearance Committee on Human Rights Related to Research Involving Human Subjects, Faculty of Medicine, Ramathibodi Hospital, Mahidol University (MURA2019/769).

***Sample preparation***

The preparation of human urine samples was performed based on the method previously described with slight modification [37]. The urine samples were stored at −80 °C prior to the extraction. A total of 100 µL of pooled urine samples (*n* = 3) were diluted with 900 µL of acetonitrile:methanol (1:1, *V*/*V*), vortexed for 30 s, sonicated for 10 min, and stored at −20 °C for one hour to facilitate protein precipitation. Subsequently, the samples were centrifuged at 13,000 *g* for 15 min. The 900 µL of supernatant was collected, and kept at −80 °C until the analysis.

**UPLC/TWIMS-QTOF-MS analysis**

Standards and samples were chromatographically separated by UPLC (ACQUITY UPLC, Waters, Milford, MA, USA) using an ACQUITY BEH HILIC column (2.1 mm × 100 mm, 1.7 µm; Waters, Milford, MA, USA). Separate acidic and basic chromatographic methods were developed for positive and negative electrospray ionization respectively, to accommodate the analysis of the analytes with a wide range of polarities. For all methods, mobile phase A consisted of acetonitrile:water (95:5, *V*/*V*) and mobile phase B acetonitrile:water (50:50, *V*/*V*). For acidic conditions, both mobile phases contained 10 mM ammonium formate and 0.125% formic acid (pH 3.0), and for basic conditions, both contained 10 mM ammonium acetate and 0.04% ammonium hydroxide (pH 9.0). An injection volume of 5 µL and column temperature of 45 °C were used in both conditions. The chromatographic gradient was as follows: 0−1 min, 99% A; 1−10 min, 99%−65% A; 10−12 min, 65%−40% A; 12−15 min 40% A; and 15−20 min, re-equilibration at 99% A, with a flow rate of 0.4 mL/min.

The ACQUITY UPLC system was coupled to a Synapt G2-Si hybrid quadrupole/traveling wave ion mobility/time-of-flight mass spectrometer (Waters, Manchester, UK) with an electrospray ionization (ESI) source. In the positive ESI mode (ESI^+^), the following parameters were applied: capillary voltage 2.5 kV; cone voltage 30 V; source temperature 100 °C; and desolvation temperature 200 °C. In the negative ESI mode (ESI^−^), the following conditions were used: capillary voltage 2.0 kV; cone voltage 40 V; source temperature 100 °C; and desolvation temperature 200 °C. For the TWIMS settings, the optimized parameters for the ESI^+^ mode were as follows: nitrogen flow rate 90 mL/min; WV 800 m/s; WH 30 V; trap bias 35 V; and helium bias 30 V. For the ESI^−^ mode, the following optimized parameters were used: nitrogen flow rate at 90 mL/min; WV 1,000 m/s; WH 30 V; trap bias at 35 V; and helium bias at 30 V. The TWIMS-QTOF-MS was mass and CCS calibrated using Waters Major Mix IMS/TOF Calibration solution, and real time single point calibration correction was performed using leucine enkephalin as reference LockMass and LockCCS to maintain mass and CCS accuracy during the long analytical acquisitions.

Data was collected using the data independent high definition MS^E^ (HDMS^E^) mode with a 0.2 s scan time, and mass range, *m*/*z* 50−1,000. In the HDMS^E^ mode, the quadrupole is non-selective (RF only), and the collision energy (CE) alternates between a low-energy function (CE 4 eV) to monitor intact precursor ions, and high-energy function (CE ramp 20–40 eV) to observe the dissociation product ions. Argon (> 99.999%) was used as a collision gas for collision-induced dissociation (CID). The TOF analyzer was operated in the sensitivity mode, which provided a resolving power of approximately 10,000 full width at half-maximum (FWHM).

**Precision, accuracy, and matrix effect on TWIMS-derived CCS values**

To determine the precision of the TWIMS-derived CCS values (^TW^CCS_N2_), the experiments were carried out using a set of representative standard metabolites (*n* = 45) that has been previously characterized using drift tube ion mobility mass spectrometry (DTIMS) and TWIMS instruments [12, 22]. The representative metabolites were analyzed individually over three days, and calculated as the percentage of relative standard deviation (%RSD). For accuracy determination, the differences between the ^TW^CCS_N2_ values derived from this study and CCS values previously reported using DTIMS or TWIMS were calculated as a CCS difference error (ΔCCS%) [15], as expressed in equation 1, where CCS_B_ represented to literature CCS values and CCS_A_ represented to measured CCS values in this study

$$\text{∆CCS\% =}\frac{\text{CCS}\text{B}-\text{CCS}\text{A}}{\text{average CCS}\text{A,B}}\text{×100}\text{ }\text{ }\text{(1) }$$

The influence of matrix effects on the CCS values was investigated by spiking human urine (900 µL) with reference standards representing a variety molecular class (*n* = 62) at a concentration of 50 µM (analyzed in ESI^−^ mode). The obtained CCS values of the metabolites in human urine were then compared with those in neat solvent.

**Data processing and analysis**

Mass spectra and mobility spectra were processed using MassLynx V4.1 and DriftScope V2.9 (Waters, USA), respectively. Metabolite identifications were performed using Progenesis QI (Nonlinear Dynamics, Newcastle, UK). Briefly, each HDMS^E^ data file is LockMass and LockCCS corrected, and all low and high energy features are retention and drift time aligned for that injection during date import, and *m/z* vs RT ion-intensity maps are generated for RT alignment and co-mapping of features between runs. After alignment, components are detected, grouping together their isotopologue peaks, thus allowing their isotopic distributions and charge states to be determined, whilst separating overlapping components. Once the peak features have been peak picked, the number of components can be further reduced by searching for and grouping together adducts for those components. The adduct ions [M+H]^+^, [M+Na]^+^, [M+H−H_2_O]^+^, and [M]^+^ were selected for ESI^+^, and [M−H]^−^ and [M+Na−2H]^−^ for ESI^−^ in this analysis. If multiple adducts can be identified for a component, the neutral mass can be determined, and the adducts cannot be assigned as separate components. Adduct selection therefore affects the number of identified compounds and the accuracy of metabolite identification.

All putative compound identifications were made using Progenesis QI MetaScope, searching against the HMDB structural database (SDF) version 5.0 [38]. The matching score (0−100) was calculated based on five parameters (each contributing 20 towards the final score); mass accuracy of precursor ions, isotope pattern similarity, closeness of RT and CCS values, and the peaks matched between the experimental MS/MS spectra and either fragment ion libraries created using reference standards, or theoretical *in silico* fragments calculated from the chemical structures. A higher score indicates a higher confidence in compound identification. For screening of metabolites in human urine, the search criteria used were as follows; precursor and product ion mass error tolerances ±20 ppm, isotope similarity ≥ 80%, RT tolerance ±0.3 min, and ΔCCS ±2% or ±4 Å^2^ to balance filter efficiency and avoid over-filtering. The HDMS^E^ RAW data files of human urine analyzed in ESI^+^ and ESI^−^ were deposited at the Mass Spectrometry Interactive Virtual Environment (MassIVE) under the accession number MSV000090370.

**Database implementation**

Siriraj Metabolomics Data Warehouse (SiMD) was built to collect the 174 metabolites and acquired properties. The database is running on MariaDB server 10.4.20 (https://mariadb.org/). The web interface was implemented with a standard HyperText Markup Language (HTML), and JavaScript programming language cooperating with PHP 7.4.21 (https://www.php.net/) for data queries.

# **Supplementary results**


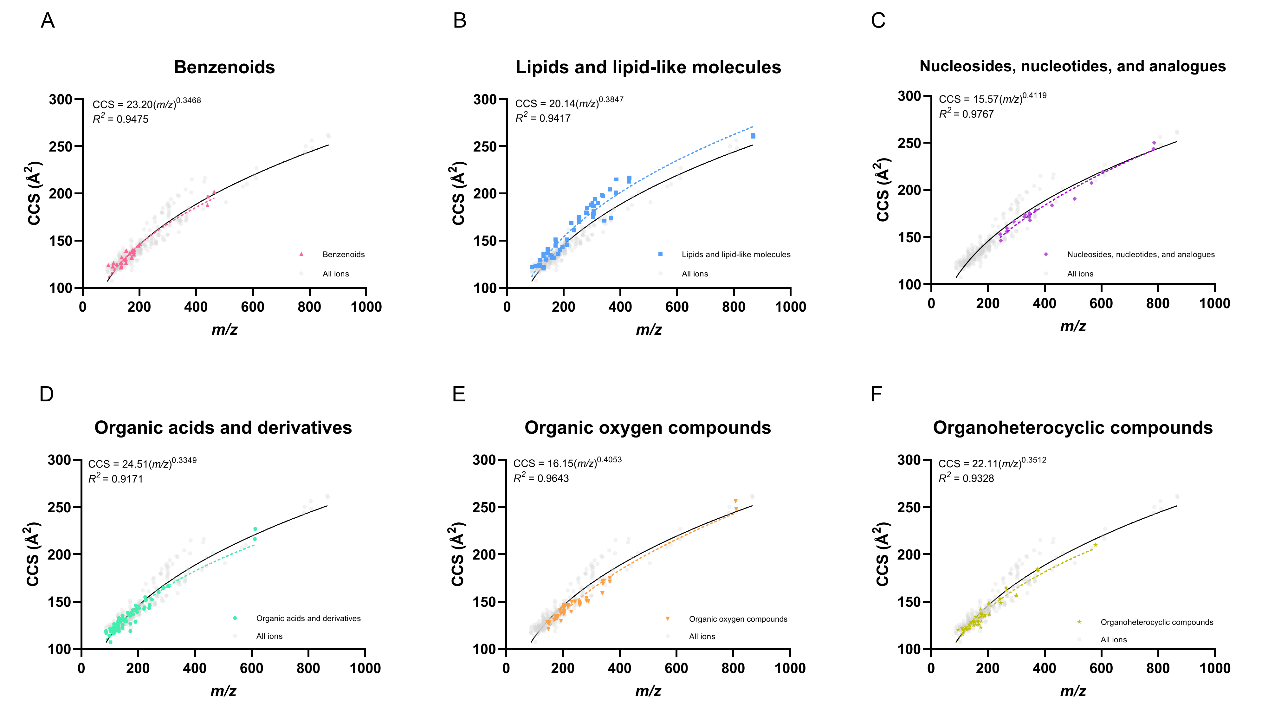


**Fig. S1.** Correlations between *m/z* and CCS values of the studied compound categorized by super classes: (A) Benzenoids (*R*^2^ = 0.9475), (B) Lipids and lipid-like molecules (*R*^2^ = 0.9417), (C) Nucleosides, nucleotides, and analogues (*R*^2^ = 0.9767), (D) Organic acids and derivatives (*R*^2^ = 0.9171), (E) Organic oxygen compounds (*R*^2^ = 0.9643), and (F) Organoheterocyclic compounds (*R*^2^ = 0.9328). The black solid line indicates the main trendline of all studied ions and the colored dashed line represents the trendline within each super classes.


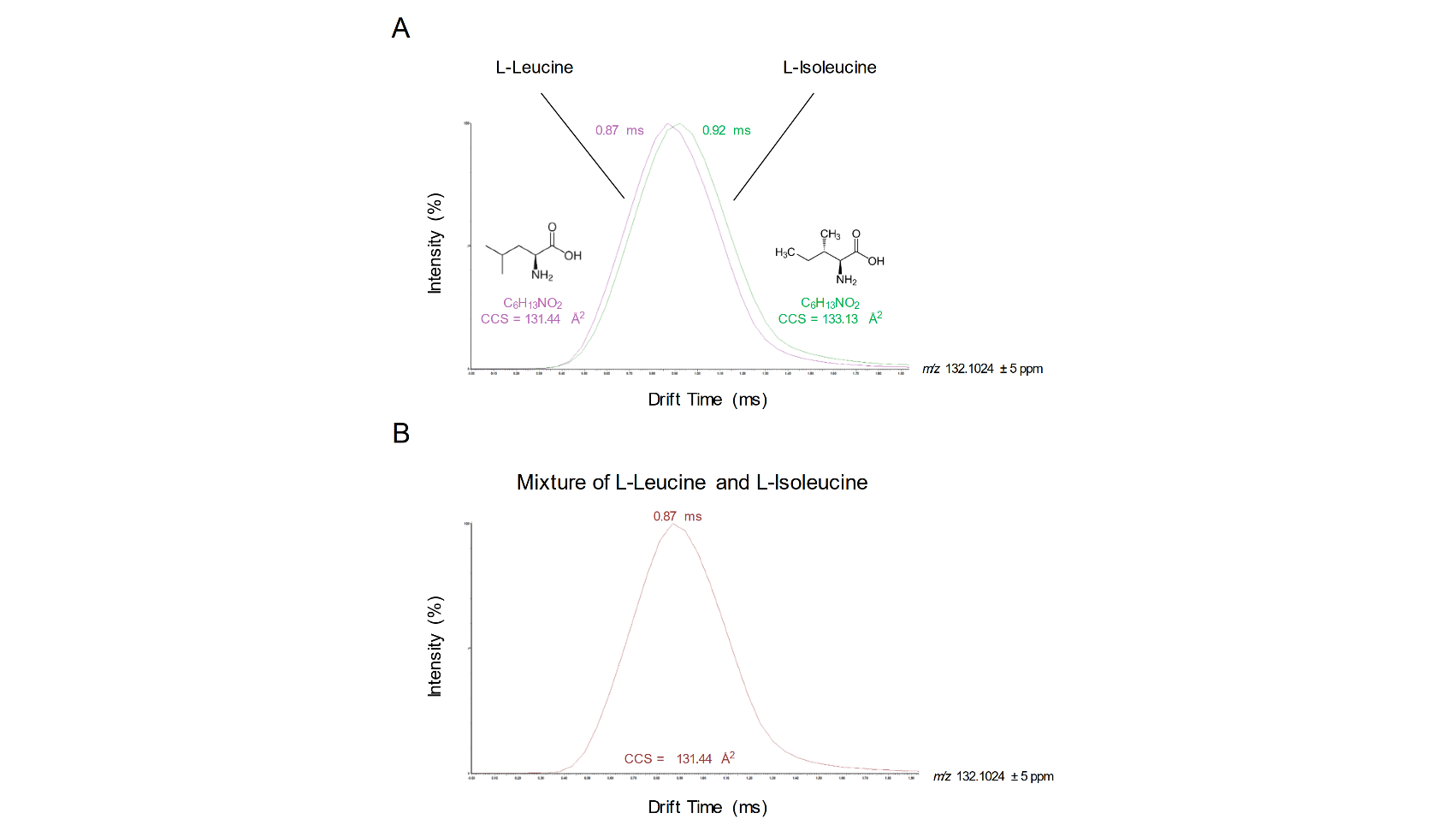


**Fig. S2.** (A) The overlaid of extracted ion mobiligram at *m*/*z* 132.1024 ± 5 ppm for [M+H]^+^ ions of structural isomers L-leucine and L-isoleucine. (B) The extracted ion mobiligram of the mixture of L-leucine and L-isoleucine.


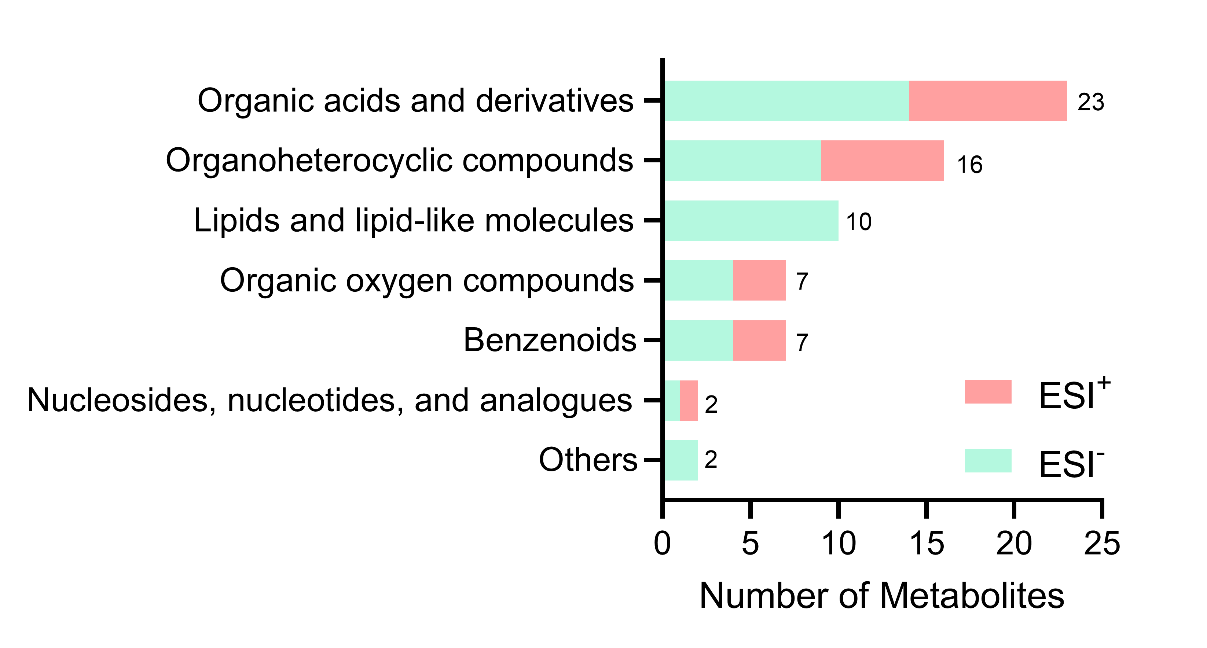


## **Fig. S3.** Level 1 metabolite annotation in district chemical super classes identified in human urine under positive electrospray ionization (ESI^+^) and negative electrospray ionization (ESI^−^) modes.


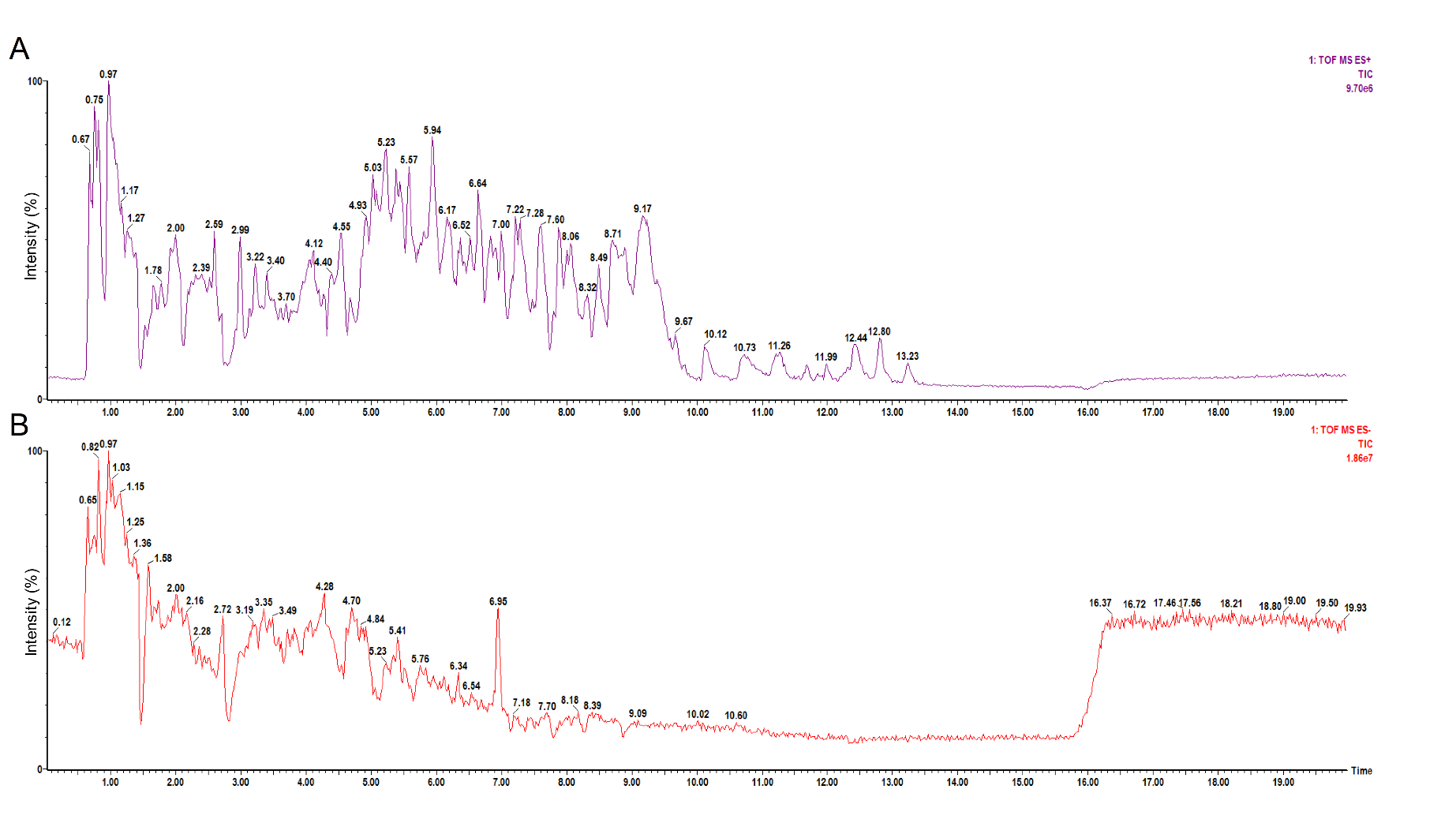


##

## **F****ig. S4.** Total ion chromatogram (TIC) of human urine analyzed in (A) positive electrospray ionization (ESI^+^) and (B) negative electrospray ionization (ESI^−^) modes.


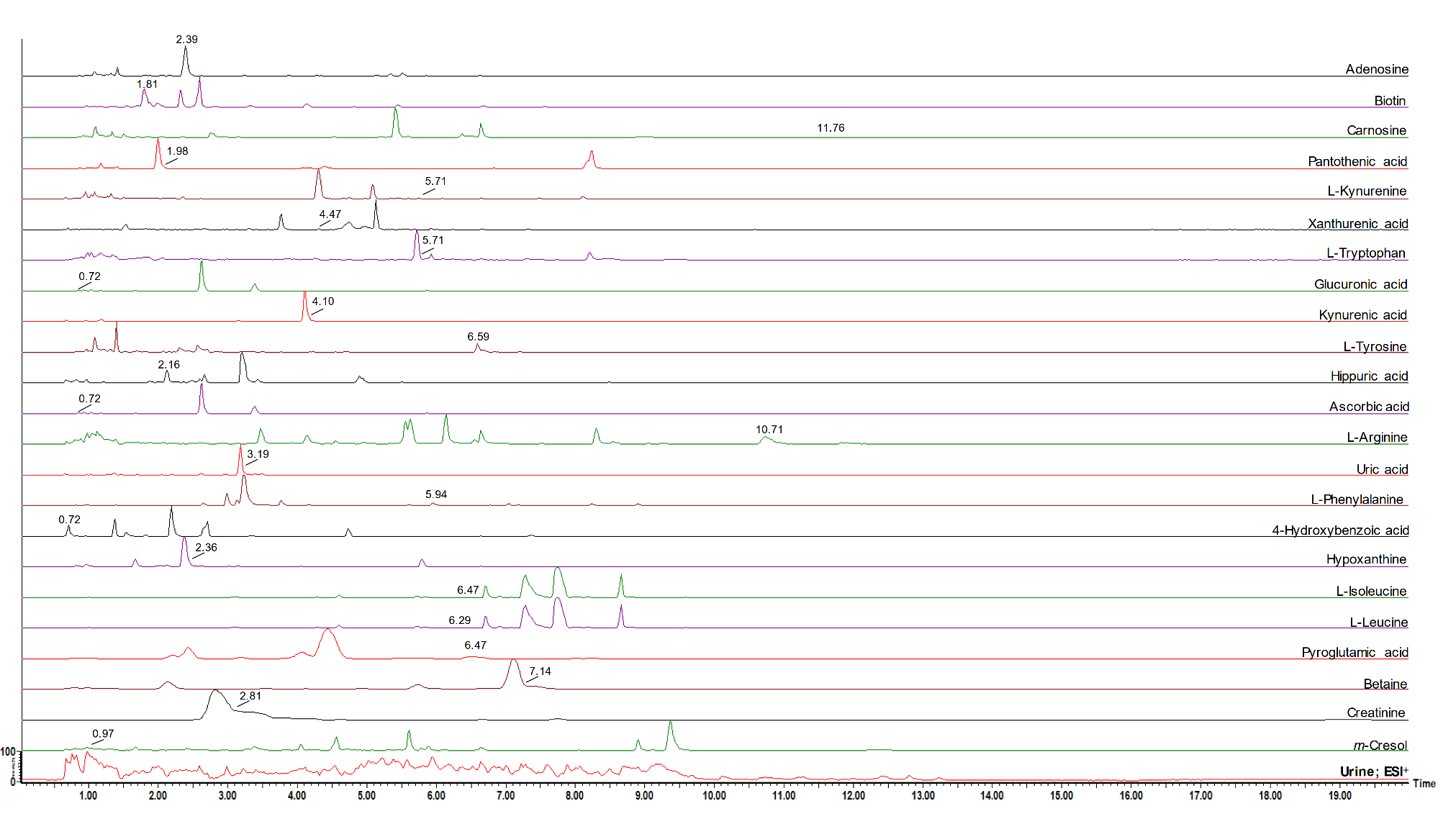


## **F****ig. S5.** Extract ion chromatogram (XIC) of metabolites identified in human urine analyzed under positive electrospray ionization (ESI^+^) mode.


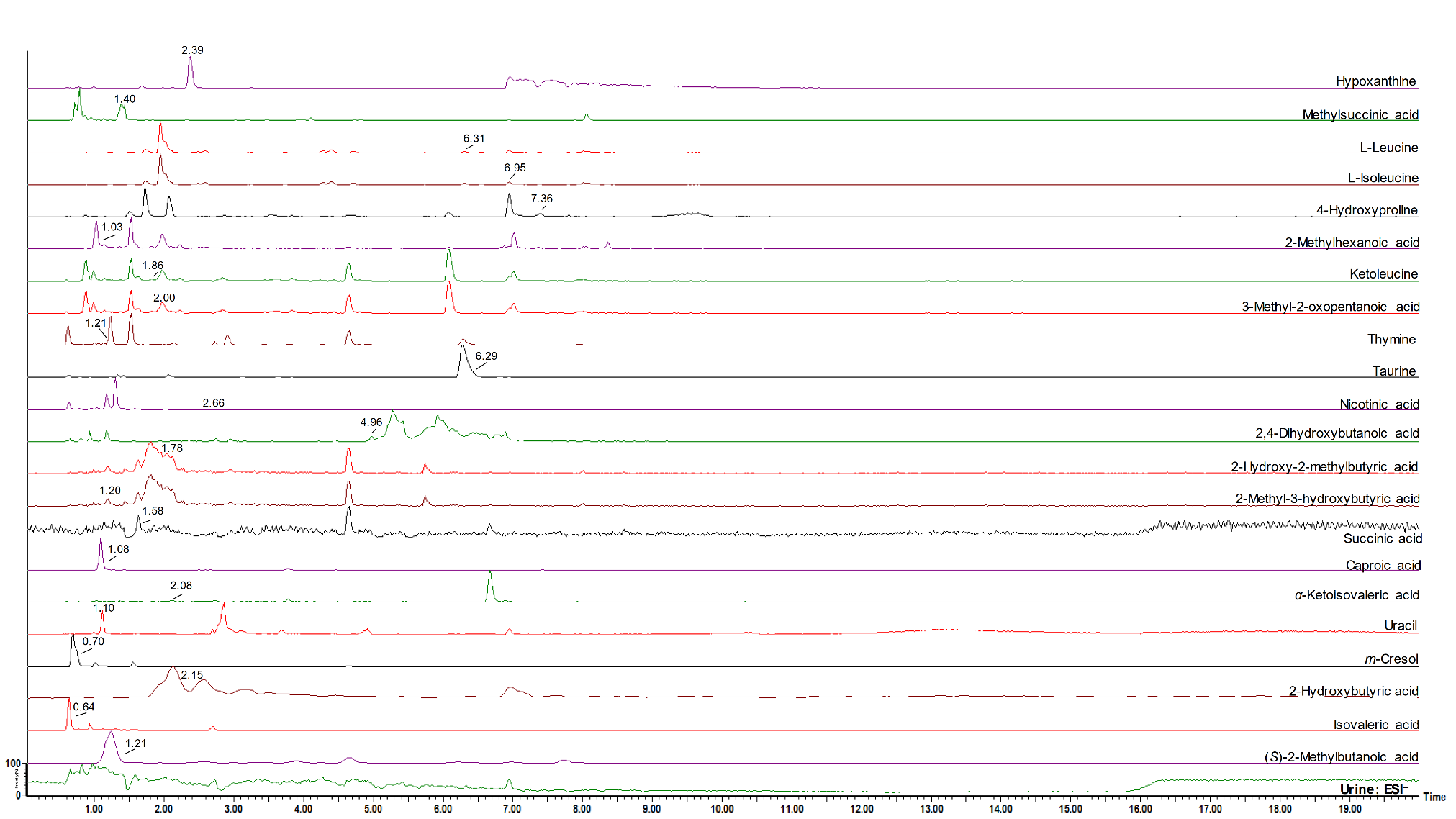


**F****ig. S6.** Extract ion chromatogram (XIC) of metabolites identified in human urine analyzed under negative electrospray ionization (ESI^−^) mode.

##
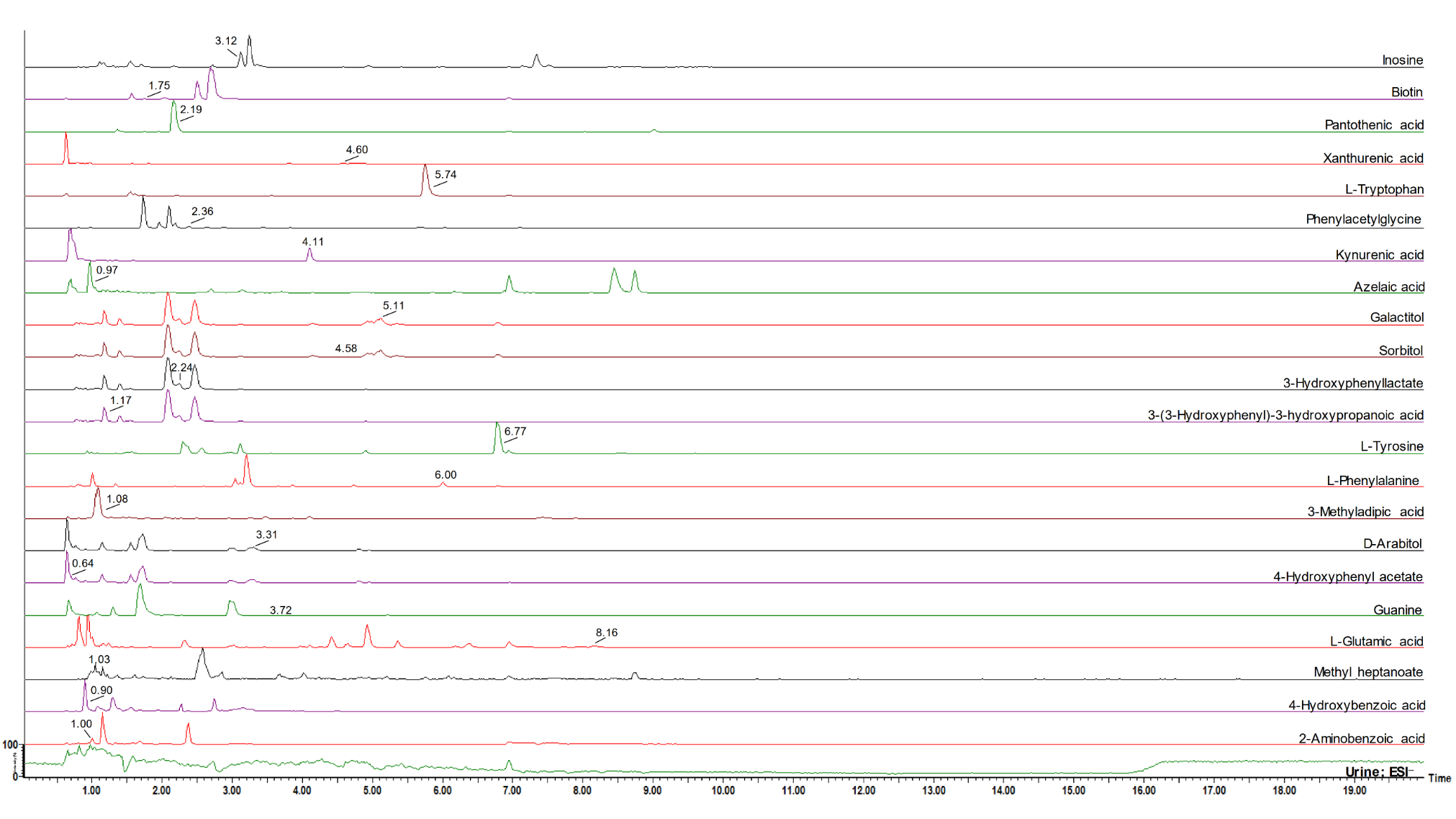


##

## **F****ig. S6.** Extract ion chromatogram (XIC) of metabolites identified in human urine analyzed under negative electrospray ionization (ESI^−^) mode (cont.).

## **Table S2.** Representative very low-molecular weight metabolites (VLMs) used for optimizing the traveling wave ion mobility spectrometry (TWIMS) parameters in this study.

| No. | Compound | Molecular formular | Monoisotopic mass (Da) | Adduct type | *m*/*z* |
| --- | --- | --- | --- | --- | --- |
| 1 | Butyric acid | C_4_H_8_O_2_ | 88.0524 | [M−H]^−^ | 87.0446 |
| 2 | L-alanine | C_3_H_7_NO_2_ | 89.0476 | [M−H]^−^ | 88.0383 |
| 3 | Lactic acid | C_3_H_6_O_3_ | 90.0316 | [M−H]^−^ | 89.0234 |
| 4 | Isovaleric acid | C_5_H_10_O_2_ | 102.0680 | [M−H]^−^ | 101.0602 |
| 5 | L-serine | C_3_H_7_NO_3_ | 105.0425 | [M−H]^−^ | 104.0345 |
| 6 | Creatinine | C_4_H_7_N_3_O | 113.0589 | [M−H]^−^ | 112.0511 |
| 7 | Nicotinic acid | C_6_H_5_NO_2_ | 123.0320 | [M−H]^−^ | 122.0242 |
| 8 | Ornithine | C_5_H_12_N_2_O_2_ | 132.0898 | [M−H]^−^ | 131.0807 |
| 9 | L-phenylalanine | C_9_H_11_NO_2_ | 165.0789 | [M−H]^−^ | 164.0709 |
| 10 | L-arginine | C_6_H_14_N_4_O_2_ | 174.1116 | [M−H]^−^ | 173.1024 |
| 11 | Citrulline | C_6_H_13_N_3_O_3_ | 175.0956 | [M−H]^−^ | 174.0873 |
| 12 | L-tryptophan | C_11_H_12_N_2_O_2_ | 204.0898 | [M−H]^−^ | 203.0832 |
| 13 | Carnosine | C_9_H_14_N_4_O_3_ | 226.1065 | [M−H]^−^ | 225.0991 |
| 14 | Palmitic acid | C_16_H_32_O_2_ | 256.2402 | [M−H]^−^ | 255.2334 |
| 15 | Eicosapentanoic acid | C_20_H_30_O_2_ | 302.2245 | [M−H]^−^ | 301.2173 |
| 16 | Behenic acid | C_22_H_44_O_2_ | 340.3341 | [M−H]^−^ | 339.3281 |
| 17 | *α*-tocopherol | C_29_H_50_O_2_ | 430.3810 | [M−H]^−^ | 429.3727 |
| 18 | ATP | C_10_H_16_N_5_O_13_P_3_ | 506.9957 | [M−H]^−^ | 505.9869 |
| 19 | UDPAG | C_17_H_27_N_3_O_17_P_2_ | 607.0815 | [M−H]^−^ | 606.0757 |
| 20 | FAD | C_27_H_33_N_9_O_15_P_2_ | 785.1571 | [M−H]^−^ | 784.1516 |

## **Table S4.** Inter-day precision (*n* = 3) for 45 representative metabolites.

| No. | Compound | HMDB ID | Monoisotopic mass (Da) | *m*/*z* | Adduct | Average CCS (Å^2^) | RSD% (*n* = 3) |
| --- | --- | --- | --- | --- | --- | --- | --- |
| 1 | Isobutyric acid | HMDB01873 | 88.0524 | 87.0437 | [M−H]^−^ | 118.84 | 0.813327553 |
| 2 | L-alanine | HMDB00161 | 89.0476 | 88.0383 | [M−H]^−^ | 117.06 | 0 |
| 3 | Lactic acid | HMDB00190 | 90.0316 | 89.0234 | [M−H]^−^ | 116.91 | 0.854328063 |
| 4 | Phosphoric acid | HMDB02142 | 97.9768 | 98.9847 | [M+H]^+^ | 125.92 | 0.721421489 |
| 5 | *α*-hydroxyisobutyric acid | HMDB00729 | 104.0473 | 103.0389 | [M−H]^−^ | 119.98 | 0 |
| 6 | Succinic acid | HMDB00254 | 118.0266 | 117.0183 | [M−H]^−^ | 118.44 | 0.797703570 |
| 7 | Nicotinic acid | HMDB01488 | 123.0320 | 124.0409 | [M+H]^+^ | 122.92 | 0.697360000 |
| 8 | Thymine | HMDB00262 | 126.0429 | 125.0346 | [M−H]^−^ | 120.89 | 0.762040620 |
| 9 | Hydroxyproline | HMDB00725 | 131.0582 | 132.0661 | [M+H]^+^ | 125.85 | 0.668079268 |
| 10 | L-leucine | HMDB00687 | 131.0946 | 132.1019 | [M+H]^+^ | 132.88 | 0 |
| 11 | Malic acid | HMDB00156 | 134.0215 | 133.0122 | [M−H]^−^ | 118.53 | 1.47x10^−14^ |
| 12 | Adenine | HMDB00034 | 135.0544 | 134.0477 | [M−H]^−^ | 126.29 | 0.693904837 |
| 13 | *α*-ketoglutaric acid | HMDB00208 | 146.0215 | 145.0124 | [M−H]^−^ | 122.30 | 0 |
| 14 | L-lysine | HMDB00182 | 146.1055 | 147.1138 | [M+H]^+^ | 131.69 | 0 |
| 15 | Dopamine | HMDB00073 | 153.0789 | 154.0865 | [M+H]^+^ | 139.55 | 0 |
| 16 | L-histidine | HMDB00177 | 155.0695 | 156.0776 | [M+H]^+^ | 131.08 | 0 |
| 17 | L-carnitine | HMDB00062 | 161.1051 | 162.1125 | [M+H]^+^ | 137.38 | 0.621096446 |
| 18 | 3-methylglutaconic acid | HMDB00522 | 144.0422 | 165.0161 | [M+Na-2H] ^−^ | 131.48 | 0 |
| 19 | L-phenylalanine | HMDB00159 | 165.0790 | 166.0868 | [M+H]^+^ | 137.14 | 0.592533952 |
| 20 | Uric acid | HMDB00289 | 168.0283 | 169.0358 | [M+H]^+^ | 133.36 | 0.634387181 |
| 21 | *cis*-aconitic | HMDB00072 | 174.0164 | 173.0079 | [M−H]^−^ | 126.73 | 0 |
| 22 | D-xylose | HMDB00098 | 150.0528 | 173.0419 | [M+Na]^+^ | 131.78 | 0 |
| 23 | Ribitol | HMDB00508 | 152.0684 | 175.0575 | [M+Na]^+^ | 133.34 | 0.611480734 |
| 24 | Ascorbic acid | HMDB00044 | 176.0320 | 177.0394 | [M+H]^+^ | 134.39 | 0 |
| 25 | Hippuric acid | HMDB00714 | 179.0582 | 178.0514 | [M−H]^−^ | 139.07 | 0 |

| No. | Compound | HMDB ID | Monoisotopic mass (Da) | *m*/*z* | Adduct | Average CCS (Å^2^) | RSD% (*n* = 3) |
| --- | --- | --- | --- | --- | --- | --- | --- |
| 26 | L-tyrosine | HMDB00158 | 181.0738 | 182.0824 | [M+H]^+^ | 141.09 | 0 |
| 27 | Azelaic acid | HMDB00784 | 188.1048 | 187.0980 | [M−H]^−^ | 139.96 | 0.519140029 |
| 28 | Citric acid | HMDB00094 | 192.0270 | 191.0145 | [M−H]^−^ | 128.76 | 0 |
| 29 | D-fructose | HMDB00660 | 180.0633 | 203.0530 | [M+Na]^+^ | 140.11 | 0 |
| 30 | D-glucose | HMDB00122 | 180.0633 | 203.0537 | [M+Na]^+^ | 143.24 | 0 |
| 31 | D-galactose | HMDB00143 | 180.0633 | 203.0539 | [M+Na]^+^ | 138.53 | 0.582174390 |
| 32 | myo-inositol | HMDB00211 | 180.0633 | 203.0539 | [M+Na]^+^ | 146.00 | 0.561191148 |
| 33 | L-kynurenine | HMDB00684 | 208.0847 | 209.0924 | [M+H]^+^ | 146.07 | 0 |
| 34 | Galactose 1-phosphate | HMDB00645 | 260.0297 | 259.0215 | [M−H]^−^ | 147.62 | 0.486332820 |
| 35 | Glucose 6-phosphate | HMDB01401 | 260.0297 | 259.0225 | [M−H]^−^ | 154.07 | 0.479599616 |
| 36 | Fructose 6-phosphate | HMDB00124 | 260.0297 | 259.0226 | [M−H]^−^ | 151.18 | 0 |
| 37 | Palmitic acid | HMDB00220 | 256.2402 | 279.2296 | [M+Na]^+^ | 179.96 | 0 |
| 38 | Arachidonic acid | HMDB01043 | 304.2402 | 303.2332 | [M−H]^−^ | 193.88 | 0 |
| 39 | Oleic acid | HMDB00207 | 282.2558 | 305.2462 | [M+Na]^+^ | 181.57 | 0.412142022 |
| 40 | Stearic acid | HMDB00827 | 284.2715 | 307.2608 | [M+Na]^+^ | 186.65 | 0.397751132 |
| 41 | Glutathione | HMDB00125 | 307.0838 | 308.0931 | [M+H]^+^ | 160.04 | 0.483248955 |
| 42 | Arachidic acid | HMDB02212 | 312.3028 | 335.2927 | [M+Na]^+^ | 198.31 | 0.374922094 |
| 43 | Sucrose | HMDB00258 | 342.1162 | 365.1048 | [M+Na]^+^ | 171.56 | 0.446071356 |
| 44 | ADP | HMDB01341 | 427.0294 | 426.0216 | [M−H]^−^ | 183.67 | 0.319445226 |
| 45 | Acetyl CoA | HMDB01206 | 809.1257 | 808.1200 | [M−H]^−^ | 256.25 | 0 |

## **Table S4.** Inter-day precision (*n* = 3) for 45 representative metabolites (cont.).

HMDB: Human Metabolome Database; CCS: collision-cross section; RSD: relative standard deviation.

## **Table S6.** Interlaboratory comparison of CCS values.

| No. | Compound | HMDB ID | *m*/*z* | Adduct type | DTIMS_lit_^a^ | TWIMS_lit_^b^ | TWIMS^c^ | ^DT^_lit_^/TW^ΔCCS% | ^TW^_lit_^/TW^ΔCCS% |
| --- | --- | --- | --- | --- | --- | --- | --- | --- | --- |
| 1 | Uracil | HMDB00300 | 111.0184 | [M−H]^−^ | 114.0 | 115 | 115.77 | 1.54 | 0.67 |
| 2 | Creatinine | HMDB00562 | 112.0511 | [M−H]^−^ | 118.6 | − | 120.56 | 1.64 | − |
| 3 | Caproic acid | HMDB00535 | 115.0761 | [M−H]^−^ | 128.5 | − | 129.59 | 0.84 | − |
| 4 | L-valine | HMDB00883 | 116.0710 | [M−H]^−^ | 128.0 | 124 | 126.43 | −1.23 | 1.94 |
| 5 | Succinic acid | HMDB00254 | 117.0183 | [M−H]^−^ | 118.0 | 117 | 118.44 | 0.37 | 1.22 |
| 6 | Nicotinic acid | HMDB01488 | 122.0242 | [M−H]^−^ | 121.7 | 119 | 121.16 | −0.44 | 1.80 |
| 7 | Taurine | HMDB00251 | 124.0069 | [M−H]^−^ | 117.9 | 118 | 119.39 | 1.26 | 1.17 |
| 8 | Thymine | HMDB00262 | 125.0346 | [M−H]^−^ | 119.1 | − | 120.89 | 1.49 | − |
| 9 | Pyroglutamic acid | HMDB00267 | 128.0341 | [M−H]^−^ | 124.1 | − | 123.74 | −0.29 | − |
| 10 | Citraconic acid | HMDB00634 | 129.0186 | [M−H]^−^ | 119.0 | − | 120.54 | 1.29 | − |
| 11 | 3-methyl-2-oxopentanoic acid | HMDB00491 | 129.0539 | [M−H]^−^ | 127.6 | − | 126.71 | −0.70 | − |
| 12 | Hydroxyproline | HMDB00725 | 130.0509 | [M−H]^−^ | 125.0 | 125 | 126.62 | 1.29 | 1.29 |
| 13 | L-isoleucine | HMDB00172 | 130.0858 | [M−H]^−^ | 130.8 | 129 | 129.46 | −1.03 | 0.36 |
| 14 | L-leucine | HMDB00687 | 130.0860 | [M−H]^−^ | 132.6 | 129 | 131.10 | −1.14 | 1.61 |
| 15 | Methylsuccinic acid | HMDB01844 | 131.0348 | [M−H]^−^ | 124.3 | − | 121.94 | −1.92 | − |
| 16 | L-asparagine | HMDB00168 | 131.0447 | [M−H]^−^ | 124.2 | 121 | 123.48 | −0.58 | 2.03 |
| 17 | Ornithine | HMDB00214 | 131.0807 | [M−H]^−^ | 131.4 | 127 | 129.53 | −1.43 | 1.97 |
| 18 | L-aspartic acid | HMDB00191 | 132.0291 | [M−H]^−^ | 121.9 | 118 | 120.30 | −1.32 | 1.93 |
| 19 | Malic acid | HMDB00156 | 133.0122 | [M−H]^−^ | 117.4 | 117 | 118.53 | 0.96 | 1.30 |
| 20 | Hypoxanthine | HMDB00157 | 135.0323 | [M−H]^−^ | 120.6 | 118 | 121.62 | 0.84 | 3.02 |
| 21 | 2-aminobenzoic acid | HMDB01123 | 136.0393 | [M−H]^−^ | 125.8 | − | 126.13 | 0.26 | − |
| 22 | 4-hydroxybenzoic acid | HMDB00500 | 137.0239 | [M−H]^−^ | 121.1 | − | 123.01 | 1.56 | − |
| 23 | Caprylic acid | HMDB00482 | 143.1069 | [M−H]^−^ | 139.5 | − | 141.36 | 1.32 | − |
| 24 | *α*-ketoglutaric acid | HMDB00208 | 145.0124 | [M+H]^+^ | 122.4 | 121 | 122.30 | −0.08 | 1.07 |
| 25 | L-lysine | HMDB00182 | 145.0972 | [M−H]^−^ | 134.2 | 133 | 135.63 | 1.06 | 1.96 |

## **Table S6.** Interlaboratory comparison of CCS values (cont.).

| No. | Compound | HMDB ID | *m*/*z* | Adduct type | DTIMS_lit_^a^ | TWIMS_lit_^b^ | TWIMS^c^ | ^DT^_lit_^/TW^ΔCCS% | ^TW^_lit_^/TW^ΔCCS% |
| --- | --- | --- | --- | --- | --- | --- | --- | --- | --- |
| 26 | L-glutamic acid | HMDB00148 | 146.0444 | [M−H]^−^ | 125.3 | 123 | 125.39 | 0.07 | 1.92 |
| 27 | L-methionine | HMDB00696 | 148.0432 | [M−H]^−^ | 132.3 | 128 | 132.57 | 0.20 | 3.51 |
| 28 | 3-hydroxyanthranilic acid | HMDB01476 | 152.0342 | [M−H]^−^ | 128.9 | − | 126.47 | −1.90 | − |
| 29 | Dopamine | HMDB00073 | 152.0718 | [M−H]^−^ | 132.4 | − | 130.85 | −1.18 | − |
| 30 | Gentisic acid | HMDB00152 | 153.0194 | [M−H]^−^ | 124.5 | − | 126.41 | 1.52 | − |
| 31 | L-histidine | HMDB00177 | 154.0614 | [M−H]^−^ | 128.5 | 127 | 129.28 | 0.61 | 1.78 |
| 32 | Allantoin | HMDB00462 | 157.0350 | [M−H]^−^ | 126.8 | 128 | 126.16 | −0.51 | -1.45 |
| 33 | Oxoadipic acid | HMDB00225 | 159.0284 | [M−H]^−^ | 127.0 | − | 128.96 | 1.53 | − |
| 34 | L-phenylalanine | HMDB00159 | 164.0709 | [M−H]^−^ | 141.0 | 136 | 138.51 | −1.78 | 1.83 |
| 35 | Quinolinic acid | HMDB00232 | 166.0192 | [M−H]^−^ | 128.1 | − | 128.56 | 0.36 | − |
| 36 | Uric acid | HMDB00289 | 167.0214 | [M−H]^−^ | 125.7 | 123 | 125.59 | −0.09 | 2.08 |
| 37 | L-arginine | HMDB00517 | 173.1024 | [M−H]^−^ | 136.6 | 134 | 136.62 | 0.01 | 1.94 |
| 38 | Citrulline | HMDB00904 | 174.0873 | [M−H]^−^ | 135.6 | 133 | 135.18 | −0.31 | 1.63 |
| 39 | D-mannose | HMDB00169 | 179.0543 | [M−H]^−^ | − | 130 | 132.14 | − | 1.63 |
| 40 | L-tyrosine | HMDB00158 | 180.0664 | [M−H]^−^ | 143.7 | 139 | 141.65 | −1.44 | 1.89 |
| 41 | 3-methyladipic acid | HMDB00555 | 181.0472 | [M+Na−2H]^−^ | 134.1 | − | 135.93 | 1.36 | − |
| 42 | Sorbitol | HMDB00247 | 181.0722 | [M−H]^−^ | 131.4 | − | 133.44 | 1.54 | − |
| 43 | Epinephrine | HMDB00068 | 182.0809 | [M−H]^−^ | 140.9 | − | 140.21 | −0.49 | − |
| 44 | Azelaic acid | HMDB00784 | 187.0980 | [M−H]^−^ | 139.3 | − | 139.96 | 0.47 | − |
| 45 | Kynurenic acid | HMDB00715 | 188.0334 | [M−H]^−^ | 134.4 | − | 134.49 | 0.07 | − |
| 46 | Citric acid | HMDB00094 | 191.0145 | [M−H]^−^ | 126.6 | 127 | 128.76 | 1.69 | 1.38 |
| 47 | Phenylacetylglycine | HMDB00821 | 192.0669 | [M−H]^−^ | 147.2 | − | 146.28 | −0.63 | − |
| 48 | Glucuronic acid | HMDB00127 | 193.0354 | [M−H]^−^ | 129.2 | 130 | 129.91 | 0.55 | -0.07 |
| 49 | Xanthurenic acid | HMDB00881 | 204.0293 | [M−H]^−^ | 134.1 | − | 136.52 | 1.79 | − |
| 50 | Pantothenic acid | HMDB00210 | 218.1033 | [M−H]^−^ | 147.7 | 143 | 146.44 | −0.86 | 2.38 |

## **Table S6.** Interlaboratory comparison of CCS values (cont.).

| No. | Compound | HMDB ID | *m*/*z* | Adduct type | DTIMS_lit_^a^ | TWIMS_lit_^b^ | TWIMS^c^ | ^DT^_lit_^/TW^ΔCCS% | ^TW^_lit_^/TW^ΔCCS% |
| --- | --- | --- | --- | --- | --- | --- | --- | --- | --- |
| 51 | L-cystathionine | HMDB00099 | 221.0598 | [M−H]^−^ | 142.1 | − | 142.48 | 0.27 | − |
| 52 | Carnosine | HMDB00033 | 225.0991 | [M−H]^−^ | 152.3 | − | 154.88 | 1.68 | − |
| 53 | D-ribose 5-phosphate | HMDB01548 | 229.0111 | [M−H]^−^ | 139.4 | 137 | 139.60 | 0.14 | 1.88 |
| 54 | Uridine | HMDB00296 | 243.0619 | [M−H]^−^ | 151.2 | 146 | 153.03 | 1.20 | 4.70 |
| 55 | Biotin | HMDB00030 | 243.0810 | [M−H]^−^ | 156.0 | − | 153.03 | −1.92 | − |
| 56 | Palmitic acid | HMDB00220 | 255.2334 | [M−H]^−^ | 168.5 | − | 170.14 | 0.97 | − |
| 57 | Glucose 1-phosphate | HMDB01586 | 259.0208 | [M−H]^−^ | 148.2 | − | 147.62 | −0.39 | − |
| 58 | Galactose 1-phosphate | HMDB00645 | 259.0215 | [M−H]^−^ | 147.3 | − | 147.62 | 0.22 | − |
| 59 | Glucose 6-phosphate | HMDB01401 | 259.0225 | [M−H]^−^ | 146.5 | 143 | 148.86 | 1.60 | 4.02 |
| 60 | Adenosine | HMDB00050 | 266.0881 | [M−H]^−^ | 158.2 | 151 | 155.95 | −1.43 | 3.23 |
| 61 | Inosine | HMDB00195 | 267.0730 | [M−H]^−^ | 158.8 | 153 | 159.47 | 0.42 | 4.14 |
| 62 | Arginosuccinic acid | HMDB00052 | 289.1140 | [M−H]^−^ | 165.9 | − | 164.65 | −0.76 | − |
| 63 | cAMP | HMDB00058 | 328.0452 | [M−H]^−^ | 172.1 | 167 | 171.56 | −0.31 | 2.69 |
| 64 | Sucrose | HMDB00258 | 341.1074 | [M−H]^−^ | 168.3 | 165 | 169.11 | 0.48 | 2.46 |
| 65 | AMP | HMDB00045 | 346.0549 | [M−H]^−^ | 173.1 | 169 | 175.53 | 1.39 | 3.79 |
| 66 | Riboflavin | HMDB00244 | 375.1314 | [M−H]^−^ | 186.6 | − | 184.43 | −1.17 | − |
| 67 | ADP | HMDB01341 | 426.0216 | [M−H]^−^ | 183.8 | 180 | 183.67 | −0.07 | 2.02 |
| 68 | *α*-tocopherol | HMDB01893 | 429.3727 | [M−H]^−^ | 209.6 | − | 212.59 | 1.42 | − |
| 69 | Folic acid | HMDB00121 | 440.1331 | [M−H]^−^ | 189.4 | − | 187.54 | −0.99 | − |
| 70 | ATP | HMDB00538 | 505.9869 | [M−H]^−^ | 190.3 | − | 190.77 | 0.25 | − |
| 71 | UDPG | HMDB00286 | 565.0480 | [M−H]^−^ | 205.1 | − | 207.36 | 1.10 | − |
| 72 | UDPGA | HMDB00935 | 579.0275 | [M−H]^−^ | 211.9 | − | 209.99 | −0.91 | − |
| 73 | UDPAG | HMDB00290 | 606.0757 | [M−H]^−^ | 223.1 | − | 219.62 | −1.57 | − |
| 74 | Oxidized glutathione | HMDB03337 | 611.1442 | [M−H]^−^ | 215.1 | 218 | 216.04 | 0.44 | −0.90 |

HMDB: Human Metabolome Database; CCS: collision-cross section; ^a^Picache J. et al. [22]; ^b^Paglia G.et al. [12]; ^c^This study; −: no data.

## **Table S7.** Comparison of CCS values in our library and metabolites detected in a fortified urine matrix.

| No. | Compound | HMDB ID | Monoisotopic mass (Da) | *m*/*z* | Adduct | CCS (Database)^a^ | CCS (Matrix)^b^ | ΔCCS% |
| --- | --- | --- | --- | --- | --- | --- | --- | --- |
| 1 | Isovaleric acid | HMDB00718 | 102.0680 | 101.0602 | [M−H]^−^ | 123.48 | 122.38 | −0.89 |
| 2 | *α*-hydroxyisobutyric acid | HMDB00729 | 104.0473 | 103.0389 | [M−H]^−^ | 119.98 | 119.73 | −0.21 |
| 3 | *m*-cresol | HMDB02048 | 108.0575 | 107.0493 | [M−H]^−^ | 122.73 | 120.87 | −1.53 |
| 4 | Uracil | HMDB00300 | 112.0272 | 111.0184 | [M−H]^−^ | 115.77 | 113.86 | −1.66 |
| 5 | Creatinine | HMDB00562 | 113.0589 | 112.0511 | [M−H]^−^ | 120.56 | 120.31 | −0.21 |
| 6 | 2-methyl-2-hydroxybutyric acid | HMDB01987 | 118.0629 | 117.0544 | [M−H]^−^ | 124.78 | 123.74 | −0.84 |
| 7 | Thymine | HMDB00262 | 126.0429 | 125.0346 | [M−H]^−^ | 120.89 | 120.11 | −0.65 |
| 8 | Pyroglutamic acid | HMDB00267 | 129.0425 | 128.0341 | [M−H]^−^ | 123.74 | 123.49 | −0.20 |
| 9 | Citraconic acid | HMDB00634 | 130.0266 | 129.0186 | [M−H]^−^ | 120.54 | 119.51 | −0.86 |
| 10 | 3-methyl-2-oxopentanoic acid | HMDB00491 | 130.0629 | 129.0539 | [M−H]^−^ | 126.71 | 127.95 | 0.97 |
| 11 | Ketoleucine | HMDB00695 | 130.0629 | 129.0546 | [M−H]^−^ | 128.21 | 127.95 | −0.20 |
| 12 | Hydroxyproline | HMDB00725 | 131.0582 | 130.0509 | [M−H]^−^ | 126.62 | 127.87 | 0.98 |
| 13 | Methylsuccinic acid | HMDB01844 | 132.0422 | 131.0348 | [M−H]^−^ | 121.94 | 121.69 | −0.21 |
| 14 | Hypoxanthine | HMDB00157 | 136.0385 | 135.0323 | [M−H]^−^ | 121.62 | 121.37 | −0.21 |
| 15 | 4-hydroxybenzoic acid | HMDB00500 | 138.0316 | 137.0239 | [M−H]^−^ | 123.01 | 121.22 | −1.47 |
| 16 | 3-methylglutaconic acid | HMDB00522 | 144.0422 | 165.0161 | [M+Na−2H]^−^ | 131.48 | 129.79 | −1.29 |
| 17 | L-glutamic acid | HMDB00148 | 147.0532 | 146.0444 | [M−H]^−^ | 125.39 | 125.13 | −0.21 |
| 18 | Guanine | HMDB00132 | 151.0494 | 150.0423 | [M−H]^−^ | 125.12 | 125.98 | 0.68 |
| 19 | D-arabitol | HMDB00568 | 152.0684 | 151.0597 | [M−H]^−^ | 126.54 | 126.28 | −0.21 |
| 20 | D-xylitol | HMDB02917 | 152.0684 | 151.0597 | [M−H]^−^ | 128.01 | 127.75 | −0.20 |
| 21 | Dopamine | HMDB00073 | 153.0789 | 152.0718 | [M−H]^−^ | 130.85 | 133.45 | 1.97 |
| 22 | Gentistic acid | HMDB00152 | 154.0266 | 153.0194 | [M−H]^−^ | 126.41 | 127.13 | 0.57 |
| 23 | 2,5-furandicarboxylic acid | HMDB04812 | 156.0058 | 176.9800 | [M+Na−2H]^−^ | 129.41 | 129.15 | −0.20 |

## **Table S7.** Comparison of CCS values in our library and metabolites detected in a fortified urine matrix (cont.).

| No. | Compound | HMDB ID | Monoisotopic mass (Da) | *m*/*z* | Adduct | CCS (Database)^a^ | CCS (Matrix)^b^ | ΔCCS% |
| --- | --- | --- | --- | --- | --- | --- | --- | --- |
| 24 | Oxoadipic acid | HMDB00225 | 160.0371 | 159.0284 | [M−H]^−^ | 128.96 | 127.25 | −1.33 |
| 25 | 3-methyladipic acid | HMDB00555 | 160.0735 | 159.0665 | [M−H]^−^ | 131.84 | 134.40 | 1.92 |
| 26 | Capric acid | HMDB00511 | 172.1463 | 171.1371 | [M−H]^−^ | 151.28 | 152.25 | 0.64 |
| 27 | Methyl nonanoate | HMDB31264 | 172.1463 | 171.1367 | [M−H]^−^ | 144.80 | 147.12 | 1.59 |
| 28 | Indoleacetic acid | HMDB00197 | 175.0633 | 174.0550 | [M−H]^−^ | 141.98 | 141.70 | −0.20 |
| 29 | Citrulline | HMDB00904 | 175.0956 | 174.0873 | [M−H]^−^ | 135.18 | 134.91 | −0.20 |
| 30 | Hippuric acid | HMDB00714 | 179.0582 | 178.0514 | [M−H]^−^ | 139.07 | 139.46 | 0.28 |
| 31 | D-mannose | HMDB00169 | 180.0633 | 179.0543 | [M−H]^−^ | 132.14 | 131.87 | −0.20 |
| 32 | L-tyrosine | HMDB00158 | 181.0738 | 180.0664 | [M−H]^−^ | 141.65 | 141.37 | −0.20 |
| 33 | 3-(3-hydroxyphenyl)-3-hydroxypropanoic acid | HMDB02643 | 182.0579 | 181.0500 | [M−H]^−^ | 138.92 | 140.99 | 1.48 |
| 34 | Sorbitol | HMDB00247 | 182.0790 | 181.0722 | [M−H]^−^ | 133.44 | 133.86 | 0.31 |
| 35 | Mannitol | HMDB00765 | 182.0790 | 181.0717 | [M−H]^−^ | 134.82 | 134.55 | −0.20 |
| 36 | Diethyl methylsuccinate | HMDB59814 | 188.1048 | 187.0951 | [M−H]^−^ | 139.96 | 140.12 | 0.11 |
| 37 | Kynurenic acid | HMDB00715 | 189.0425 | 188.0334 | [M−H]^−^ | 134.49 | 135.93 | 1.06 |
| 38 | Phenylacetylglycine | HMDB00821 | 193.0738 | 192.0669 | [M−H]^−^ | 146.28 | 147.60 | 0.90 |
| 39 | Vanillylmandelic acid | HMDB00291 | 198.0528 | 197.0464 | [M−H]^−^ | 144.75 | 141.86 | −2.02 |
| 40 | L-tryptophan | HMDB00929 | 204.0899 | 203.0832 | [M−H]^−^ | 148.33 | 148.88 | 0.37 |
| 41 | Xanthurenic acid | HMDB00881 | 205.0375 | 204.0293 | [M−H]^−^ | 136.52 | 136.25 | −0.20 |
| 42 | Pantothenic acid | HMDB00210 | 219.1106 | 218.1033 | [M−H]^−^ | 146.44 | 147.41 | 0.66 |
| 43 | Myristic acid | HMDB00806 | 228.2089 | 227.2021 | [M−H]^−^ | 168.91 | 166.86 | −1.22 |
| 44 | Biotin | HMDB00030 | 244.0881 | 243.0810 | [M−H]^−^ | 153.03 | 151.51 | −1.00 |
| 45 | Adenosine | HMDB00050 | 267.0967 | 266.0881 | [M−H]^−^ | 155.95 | 154.85 | −0.71 |
| 46 | Inosine | HMDB00195 | 268.0807 | 267.0730 | [M−H]^−^ | 159.47 | 160.32 | 0.53 |

## **Table S7.** Comparison of CCS values in our library and metabolites detected in a fortified urine matrix (cont.).

| No. | Compound | HMDB ID | Monoisotopic mass (Da) | *m*/*z* | Adduct | CCS (Database)^a^ | CCS (Matrix)^b^ | ΔCCS% |
| --- | --- | --- | --- | --- | --- | --- | --- | --- |
| 47 | Oleic acid | HMDB00207 | 282.2558 | 281.2472 | [M−H]^−^ | 179.19 | 177.76 | −0.80 |
| 48 | D-sedoheptulose 7-phosphate | HMDB01068 | 290.0402 | 289.0317 | [M−H]^−^ | 150.55 | 151.47 | 0.61 |
| 49 | Cinnavalininate | HMDB04078 | 300.0382 | 299.0307 | [M−H]^−^ | 156.31 | 158.34 | 1.29 |
| 50 | Eicosapentanoic acid | HMDB01999 | 302.2245 | 301.2173 | [M−H]^−^ | 190.11 | 188.70 | −0.74 |
| 51 | Arachidonic acid | HMDB01043 | 304.2402 | 303.2332 | [M−H]^−^ | 186.98 | 186.08 | −0.48 |
| 52 | Arachidic acid | HMDB02212 | 312.3028 | 311.2969 | [M−H]^−^ | 193.88 | 190.48 | −1.77 |
| 53 | cAMP | HMDB00058 | 329.0525 | 328.0452 | [M−H]^−^ | 171.56 | 173.03 | 0.85 |
| 54 | Behenic acid | HMDB00944 | 340.3341 | 339.3281 | [M−H]^−^ | 197.18 | 198.24 | 0.54 |
| 55 | Sucrose | HMDB00258 | 342.1162 | 341.1074 | [M−H]^−^ | 169.11 | 168.04 | −0.63 |
| 56 | Maltitol | HMDB02928 | 344.1318 | 343.1262 | [M−H]^−^ | 171.26 | 170.93 | −0.20 |
| 57 | cGMP | HMDB01314 | 345.0474 | 344.0402 | [M−H]^−^ | 172.34 | 171.45 | −0.52 |
| 58 | Riboflavin | HMDB00244 | 376.1382 | 375.1314 | [M−H]^−^ | 184.43 | 182.01 | −1.32 |
| 59 | ADP | HMDB01341 | 427.0294 | 426.0216 | [M−H]^−^ | 183.67 | 184.07 | 0.21 |
| 60 | *α*-tocopherol | HMDB01893 | 430.3810 | 429.3727 | [M−H]^−^ | 212.59 | 212.61 | 0.01 |
| 61 | UDPAG | HMDB00290 | 607.0815 | 606.0757 | [M−H]^−^ | 219.62 | 220.04 | 0.19 |
| 62 | Acetyl-CoA | HMDB01206 | 809.1257 | 808.1200 | [M−H]^−^ | 256.25 | 256.22 | −0.01 |

HMDB: Human Metabolome Database; CCS: collision-cross section; ^a^Measured in neat solvent; ^b^Measured in urine matrix.
